# Toward Gate-Tunable Topological Superconductivity in a Supramolecular Electron Spin Lattice

**DOI:** 10.1021/acs.nanolett.5c03396

**Published:** 2025-10-08

**Authors:** Rémy Pawlak, Jung-Ching Liu, Chao Li, Richard Hess, Hongyan Chen, Carl Drechsel, Ping Zhou, Xinyi Liu, Robert Häner, Ulrich Aschauer, Thilo Glatzel, Silvio Decurtins, Daniel Loss, Jelena Klinovaja, Shi-Xia Liu, Wulf Wulfhekel, Ernst Meyer

**Affiliations:** † Department of Physics, WSS Research Center for Molecular Quantum Systems, 27209 University of Basel, Klingelbergstrasse 82, 4056 Basel, Switzerland; ‡ Physikalisches Institut, 150232 Karlsruhe Institute of Technology, Wolfgang-Gaede-Str. 1, 76131 Karlsruhe, Germany; ¶ Department of Chemistry, Biochemistry and Pharmaceutical Sciences, W. Inäbnit Laboratory for Molecular Quantum Materials and WSS Research Center for Molecular Quantum Systems, 234685 University of Bern, Freiestrasse 3, 3012 Bern, Switzerland; § Department of Chemistry and Physics of Materials, 27257 University of Salzburg, Jakob-Haringer-Strasse 2A, 5020 Salzburg, Austria

**Keywords:** Tetraazapyrene radicals, scanning tunneling microscopy, atomic force microscopy, molecular quantum dot, Yu−Shiba−Rusinov states, topological crystalline superconductor

## Abstract

Low-dimensional magnet/superconductor hybrid systems have been proposed as a platform for achieving topological superconductivity. Here we showcase the supramolecular assembly of organic radicals directly on superconducting Pb(111), whose charge state can be controlled from anionic to neutral by the electric field of the scanning tunneling microscope. The anionic molecules obtained by an electron given by the substrate carry a spin-1/2 state and form a two-dimensional spin lattice, as confirmed by the observation of Yu–Shiba–Rusinov subgap states in tunneling spectra. At the boundary of the molecular domains, low-energy subgap states appear localized with high intensity at edges compared to the interior of the island. Tight-binding simulations suggest that their localization and spectral signatures are consistent with the emergence of topologically protected modes. Our results pave the way for the design of organic/superconductor hybrid systems with the potential to realize topological superconductivity.

## Graphical abstract

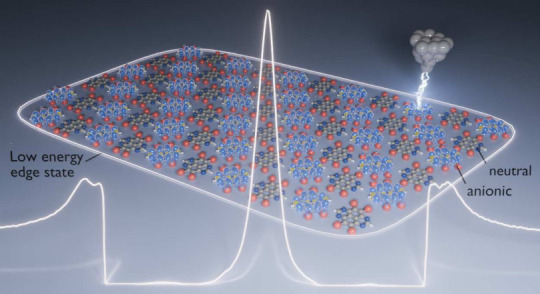

Topological superconductivity (TS) can be engineered in hybrid systems by coupling s-wave superconductors to materials like semiconducting nanowires with strong spin–orbit interaction,^1^ ferromagnetic atomic chains,^2–7^ or magnetic islands.^8–11^ A hallmark of TS is the presence of topologically protected modes at boundaries of the systems, observed as zero-energy conductance peaks in scanning tunneling spectroscopy (STS) or transport experiments. However, since disorder can close the topological gap of the nontrivial phase by severely affecting the proximitized superconducting states,^12–14^ atomic-scale measurements with high spectral resolution are essential to reliably identify these modes from conventional in-gap states.^15–18^

Low-temperature scanning tunneling microscopy (STM) with its atomic precision and spectral resolution enables the design and investigation of designer quantum materials.^19,20^ It has revealed how Yu–Shiba–Rusinov (YSR) states^21–23^ induced by magnetic impurities coupled to a superconductor depend on surface coordination,^24^ interatomic coupling,^15–18^ and magnetic anisotropy,^25^ highlighting pathways to topological phases. While dense atomic chains have been studied, dilute spin chains and two-dimensional “Shiba” lattices mediated by long-range Ruderman–Kittel–Kasuya–Yosida interaction^26^ are predicted to exhibit rich phase diagrams, including high-Chern-number TS with chiral edge states.^27–29^ Recently, topological crystalline superconductivity protected by spatial symmetries has further emerged as a promising direction,^30–32^ with STM studies showing the premises of mirror-symmetry-protected edge modes.^33^ However, a key challenge for future applications remains the local control of the chemical potential near these atomic structures with external gate voltages. Inspired by tip-induced charge-state control in quantum dots and molecules using local electric fields,^34–38^ we investigate a two-dimensional spin lattice formed by the supramolecular assembly of gate-tunable radical molecules on superconducting Pb(111). This platform offers not only a novel route to investigate spin-superconductor interactions but also to potentially discover topological crystalline superconductivity in organic-superconductor hybrid systems.

As a precursor, we used the 4,5,9,10-tetrabromo-1,3,6,8-tetraazapyrene (TBTAP) molecule (Figure 1a) consisting of an electron acceptor tetraazapyrene backbone equipped with four peripheral bromine atoms.^39^ We previously demonstrated that TBTAP^•–^ radicals maintain a 1/2 spin state on both Ag(111) and Pb(111) without a decoupling layer.^20,37,40^ For this study, TBTAP molecules were sublimed onto Pb(111) to self-assemble into large supramolecular islands (Figure 1b; see the Supporting Information). STM imaging reveals a densely packed rectangular lattice with parameters *a*_1_ = 17.2 Å and *b*_1_ = 12.3 Å (arrows in Figure 1c), showing alternating bright and dark rows. The corresponding AFM images (Figure 1d) resolve individual Br atoms as bright protrusions surrounding the TAP backbone, confirming the molecular positions in the array. Two distinct AFM contrasts are observed across different rows, labeled as charged (*c*, dashed line) and neutral (*n*, dotted line), which will be discussed later.

**Figure 1:**
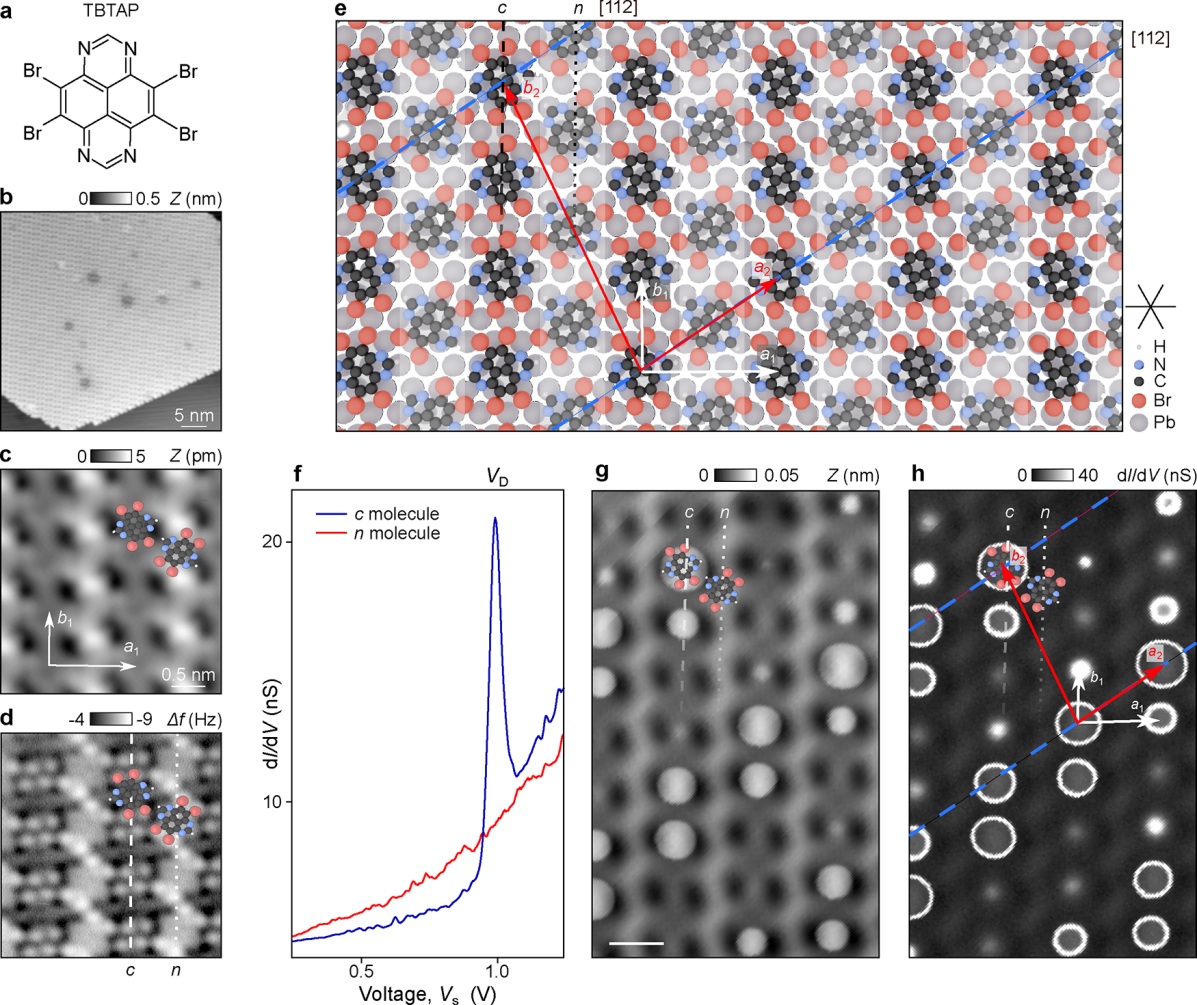
Supramolecular assembly of radical molecules on Pb(111) and their charge-state control. (a) Chemical structure of the TBTAP molecule. (b) Large-scale STM overview of a self-assembled supramolecular island on Pb(111). Imaging parameters: *V*_s_ = −0.5 V, *I*_t_ = 1 pA. (c) High-resolution STM image of the molecular lattice (*V*_s_ = 0.8 V, *I*_t_ = 0.8 pA). The unit cell is indicated by arrows. (d) Corresponding noncontact AFM image acquired with a Br-functionalized tip, revealing the alternating rows of charged (*c*) and neutral (*n*) TBTAP molecules. The overlaid ball-and-stick model identifies atoms: Br (red), N (blue), H (white), and C (black). (e) DFT-optimized model of the TBTAP supramolecular lattice on Pb(111), illustrating both the molecular (white arrows) and charge (red arrows) unit cells. Lattice parameters: *a*_1_ = 17.2 Å, *b*_1_ = 12.3Å for the molecular lattice; *a*_2_ = 20 Å, *b*_2_ = 39.9 Å for the charge modulation. Blue dashed lines indicate the [112] directions of the Pb(111) substrate. (f) d*I*/d*V* point spectra recorded on charged *c*-type (blue) and neutral *n*-type (red) TBTAP molecules. The pronounced resonance at *V*_s_ = *V*_D_ arises from tip-induced discharging of the radical. Set point parameters: *I*_t_ = 100 pA, *V*_s_ = −1.0 V. Lock-in: *f* = 611 Hz, *A*_mod_ = 20 mV. (g) STM image of the TBTAP assembly at *V*_s_ = 0.8 V, *I*_t_ = 50 pA and (h) the corresponding d*I*/d*V* map, showing tip-induced discharge rings centered around each molecule of the *c* rows.

Using density functional theory (DFT) (see the Supporting Information), we relaxed the TBTAP network on Pb(111) (Figures 1d and S1). The assembly is in relative registry with the Pb(111) surface in agreement with the experimental observations. The parameter *a*_1_ is aligned along the [110] directions, consistent with STM/AFM data: *a*_1_ follows the [110] direction as *a*_1_ = 5 × *a*_111_ = 17.5 Å and *b*_1_ aligns with the [112] direction as *b*_1_ = $2\sqrt{3}a_{111}$ = 12.1 Å, where *a*_111_ = 3.5 Å is the lattice parameter of the Pb(111) surface. Molecules are stabilized through close contact between Br atoms (C–Br···Br–C) and between Br and N atoms (C–N···Br–C). The rows marked by dashed and dotted lines correspond to charged (*c*) and neutral (*n*) molecules forming a charge superlattice, which we recently confirmed by DFT calculations.^40^ The molecules lie flat at ∼3.4 Å above the surface, suggesting that the contrast variation observed in STM/AFM images may stem from differing charge states (Figures S2–S4),^36^ but not topographic height differences or structural relaxation of the molecule (Figure S2c). Note that similar variations of STM/AFM contrasts between charge-states have been reported in previous work for tetraazapyrene molecules on Ag(111).^36,41^

Figure S2d shows a series of AFM images simulated with the probe-particle model^42^ using the Lennard-Jones force field and the Hartree potential electrostatics extracted from DFT calculations.^40^ We consider the quadrupole model for CO-tip with positive (*q*_tip_ = 0.3), negative (*q*_tip_ = −0.3) and neutral (*q*_tip_ = 0) charge-states of the tip. While a variation of the apparent bond lengths in the pyrene backbone can be observed in the simulations by Fatayer et al.,^43^ we are unable to reproduce the overall increase of contrast between charged and neutral TBTAP molecules of Figure 1e. We think that it may be related to the small magnitude of the Hartree potential and the absence of tip density of states in our simulation. Furthermore, DFT does not currently describe the superconducting state of Pb, and it is possible that the electronic structure may be missing key aspects. This point will be addressed in future experiments, which will place particular emphasis on distinguishing bond order for the different molecular charge-states using AFM imaging with CO-terminated tips.

To verify the charge-state differences, we next compared d*I*/d*V* spectra of TBTAP molecules in *c* and *n* rows (Figure 1f). Molecule *c* (blue) shows a sharp resonance at *V*_D_ ≈ 1 V, absent in molecule *n* (red). This indicates a tip-induced discharge event corresponding to a charge-state transition from anionic TBTAP^•–^ to neutral TBTAP.^35–37^ In the absence of gating by the tip electric field, TBTAP^•–^ remains in an anionic state inherited by the transfer of one electron from the substrate to the lowest unoccupied molecular orbitals (LUMO),^35,37^ which then splits into a singly unoccupied molecular orbital (SUMO) and a singly occupied molecular orbital (SOMO) (Figures S2–S4). Note that charging events expected as a dip in d*I*/d*V* spectra for negative voltages were not observed for both type of molecules. In a recent work, we also demonstrated that Δ*f*(V) spectroscopy enables to trigger the discharge of TBTAP^•–^ through capacitive coupling with the tip field, in analogy to tunneling experiments. We observed a dip in the Δ*f*(V) parabola attributed to the same charge-state transition from anionic to neutral state, as previously reported by Kocić et al.^36^ or in scanning quantum dot microscopy.^44^

Figure 1g,h shows a STM topographic image and the corresponding constant-height d*I*/d*V* map acquired at the voltage threshold *V*_s_ = *V*_D_. Bright rings centered on *c* molecules in the d*I*/d*V* map indicate the successful electron removal by gating, but also marks the precise electron location in the TBTAP assembly. The discharge can be described at first approximation by the double-junction tunneling barrier (DJTB) model,^35–37^ where the discharge efficiency is characterized by the lever arm L , which depends linearly on the tip voltage *V*_S_ and positions with respect to the molecule (Figures S5 and S6). A d*I*/d*V* cross-section acquired across *n–c–n* rows (Figure S7a) shows that discharge rings appear only at *c* rows, confirming that only TBTAP^•–^ can be discharged. The discharge event also forms in the cross-section a parabolic curve centered on the molecule, with its bottom close to 0.9 V corresponding to the minimum threshold voltage. As *V*_S_ increases, L increases linearly due to the intrinsic capacitive coupling between tip and molecule, causing the parabola to widen and explaining the voltage-dependent growth of discharge rings seen in d*I*/d*V* maps (Figures S7c–f).

At fixed *V*_s_ ≥ *V*_D_, discharging rings observed in d*I*/d*V* mapping (Figure 1h) form a superlattice with parameters *a*_2_ = 20 Å and *b*_2_ = 39.9 Å. This charge pattern is rotated by 30° relative to the molecular lattice, forming a 1×3R30° superstructure (Figure 1e). As compared to Pb(111), *a*_2_ aligns with the [112] directions (dashed blue lines), while *b*_2_ shows less commensurability with the substrate, explaining the anisotropy and 45° rotation of the Coulomb pattern. The observed variation in ring diameters between neighboring TBTAP^•–^ of charged rows further reflects a charge modulation estimated to ∼150 mV (Figure S7b). This modulation may also hint at the emergence of a charge density wave (CDW) in the organic monolayer,^45^ which will be explored in future work. For *V*_S_ ≥ 1.1 V and tip positions between adjacent molecules, discharge parabolas begin to merge, enabling the removal of two electrons (2e) from neighboring molecules (region *e* ≥ 1 in Figure S7b). As *V*_s_ increase, d*I*/d*V* maps shows expanding rings (Figure S7c) followed by their coalescence (Figure S7d). Unlike a simple superposition of rings expected for noninteracting quantum dots, the observed fusion (Figure S7e,f) indicates a cascade discharge effect between adjacent TBTAP^•–^ molecules in the *c* rows.^46^

DFT calculations confirm that radical TBTAP^•–^ on Pb(111) exhibits a spin-1/2 ground state with strong spin polarization (Figure 2a). Using tunneling spectroscopy at 35 mK with a metallic tip, we probed the YSR bound states of TBTAP^•–^ in the middle of a molecular island (Figure 2b; see the Supporting Information). Figure 2c compares d*I*/d*V* spectra of three representative TBTAP^•–^ molecules marked in Figure 2b (black spectra) with that of Pb(111) (blue) and a neutral TBTAP^0^ (red). For the last two, a hard gap centered to *E*_F_ and framed by the two coherence peaks of Pb(111)^47^ is systematically measured without in-gap states. Each TBTAP^•–^ spectrum additionally shows one pair of YSR states at energies ε_α_ = ±460 μeV, ε_β_ = ±720 μeV and ε_γ_ = ±940 μeV (dotted lines). These YSR states arise from the spin-1/2 nature of the radicals. Upon applying a 0.5 T out-of-plane magnetic field to quench superconductivity, we detected instead a Kondo resonance (Figure S8) and estimated the Kondo temperature *T*_K_ ≈ 10.3 K (Figure S9).

**Figure 2:**
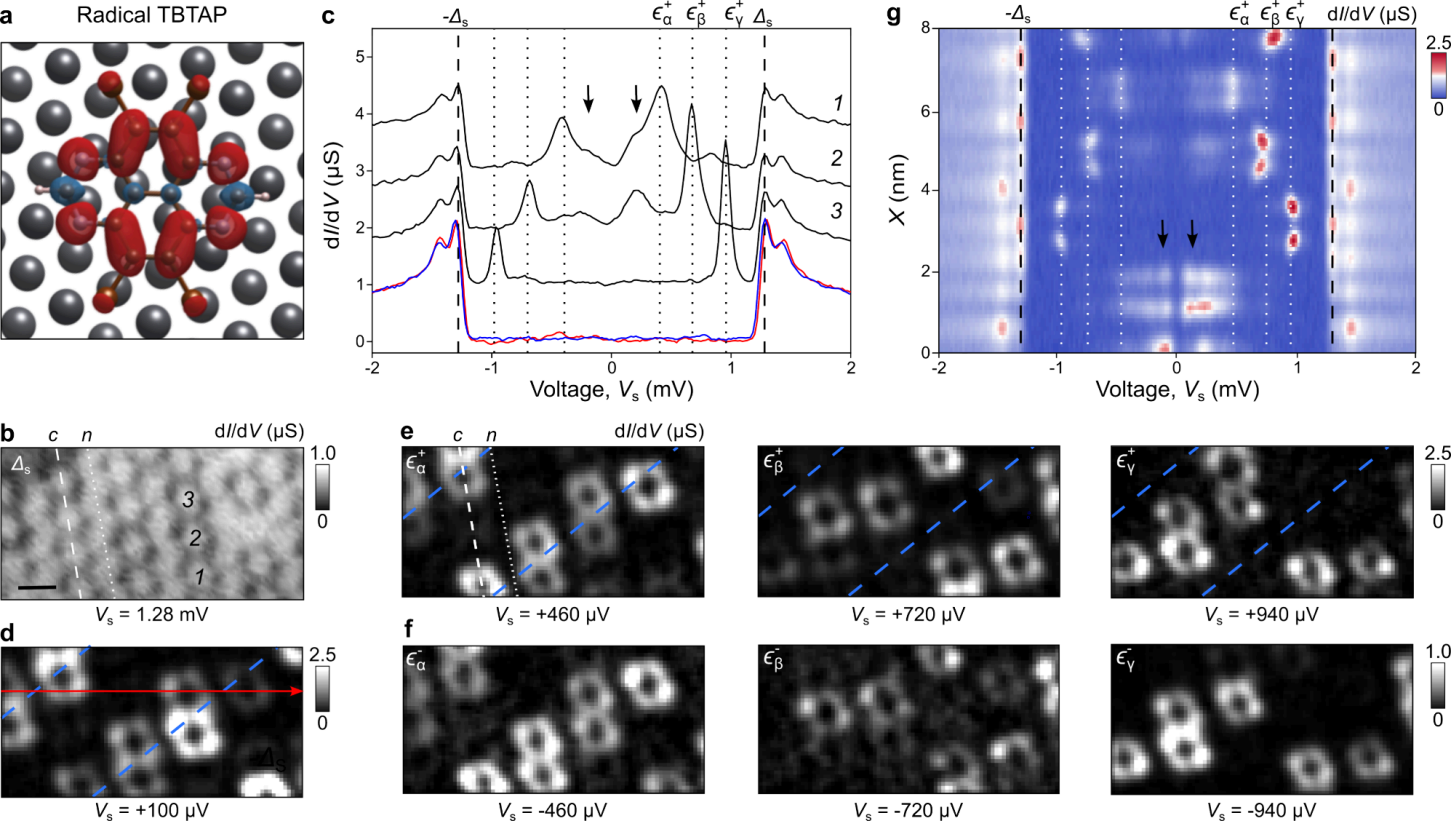
Yu–Shiba–Rusinov states of TBTAP^•–^ molecules in the center of a supramolecular island. (a) Spin density distribution of an isolated TBTAP^•–^ molecule adsorbed on Pb(111), obtained from DFT calculations. (b) d*I*/d*V* map of the TBTAP^•–^ assembly acquired at *T* = 35 mK with a metallic tip for *V*_S_ = 1.28 meV (scale bar is 1 nm, lock-in parameters: *f* = 3.28 kHz, *A*_mod_ = 40 μV, tunneling parameters: *I*_t_ = 200 pA, *V*_s_ = 5 mV). Dashed and dotted lines correspond to rows of *c* and *n* molecules, respectively. (c) d*I*/d*V* point spectra of Pb(111) (red), a neutral TBTAP molecule (blue), and three representative TBTAP^•–^ radicals (black, marked in (b)). Spectra are offset by 1 μS for clarity. The radicals exhibit YSR resonances at ε_α_ = ±460 μeV, ε_β_ = ±720 μeV, and ε_γ_ = ±940 μeV, with exact positions varying between molecules along the *c* row. Low-energy modes (LEMs) near *E*_F_ are indicated by black arrows. (d) d*I*/d*V* map at ε = 100 μeV showing spatial distribution of LEM features (blue dashed lines) and (e, f) constant-height d*I*/d*V* maps acquired at energies corresponding to ±ε_α_, ±ε_β_, and ±ε_γ_, showing the spatial profiles of the respective YSR states. Blue dashed lines in (d) and (e) indicate the [112] crystallographic directions. (g) d*I*/d*V*(*V*, *Y*) line cut recorded along the red arrow in (d). White dotted lines indicate YSR energies ±ε_α,β,γ_, while black dashed lines mark the superconducting gap edges ±Δ. Black arrows highlight the LEM features.

Differential conductance maps at energies ε_α_^±^, ε_β_^±^ and ε_γ_^±^ reveal the electron-like and hole-like YSR wave functions of TBTAP^•–^ (Figure 2e,f), showing a characteristic donut shape consistent with the calculated spin density (Figure 2a). These YSR states are confined to the molecular backbone, similar to other weakly physisorbed magnetic molecules on superconductors,^48–50^ and unlike extended states into the superconductor for chemisorbed adatoms.^8^ We also infer that the shift of the YSR states to higher energies in the assembly as compared to that of the isolated molecule (Figure S10)^20^ as well as their spatial distribution points to a coupling of the quasi-particle excitations between molecules of the supramolecular network.^18^ Figure 2g shows a d*I*/d*V* cross-section along the red line of Figure 2d, with white dotted lines marking the YSR energies ε_α,β,γ_^±^. In addition to YSR peaks, broader low-energy resonances near *E*_F_ appear (black arrows in Figure 2c,g), especially along the dashed white lines in Figure 2b,e. These low-energy modes (LEM) are also most intense at the border of the molecular island.

Using superconducting tips with a particle-hole symmetric density of states and superconducting gap Δ_t_, one expects when tunneling into a zero energy mode to observe a pair of peaks at ±Δ_t_ of equal amplitude in the d*I*/d*V* spectrum. Using Pb tips at *T* = 1 K (see the Supporting Information), the zero-energy peak thus appears shifted from zero to the finite voltages *eV* = ±Δ_t_ = 1.35 meV, while the superconducting edge is observed at ±(Δ_t_ + Δ_s_) = ±2.7 meV (Δ_s_ = 1.35 meV is the superconducting gap of the substrate). In Figure 3, spectra along a *c* row close to the island edge (Figure 3a,b) reveal shifted YSR states at ≈ 1.85–2.25 meV (Δ_t_ + ε_α, β, γ_) and sharp resonances at ±Δ_t_, consistent with the observation of the low-energy modes with superconducting tips. Constant-height d*I*/d*V* maps (Figure 3d,e) compare the spatial distribution of the hole-like wave functions near the edge extracted at ε^+^ with the LEM wave functions at +Δ_t_. While the DOS at the YSR energy is homogeneous along *c* rows, the LEM lines emerge from the ferromagnetic edge schematized in the model of Figure 3f. They propagate along the direction rotated by 60° with respect to the edge corresponding to a ferromagnetic direction of the spin structure.

**Figure 3:**
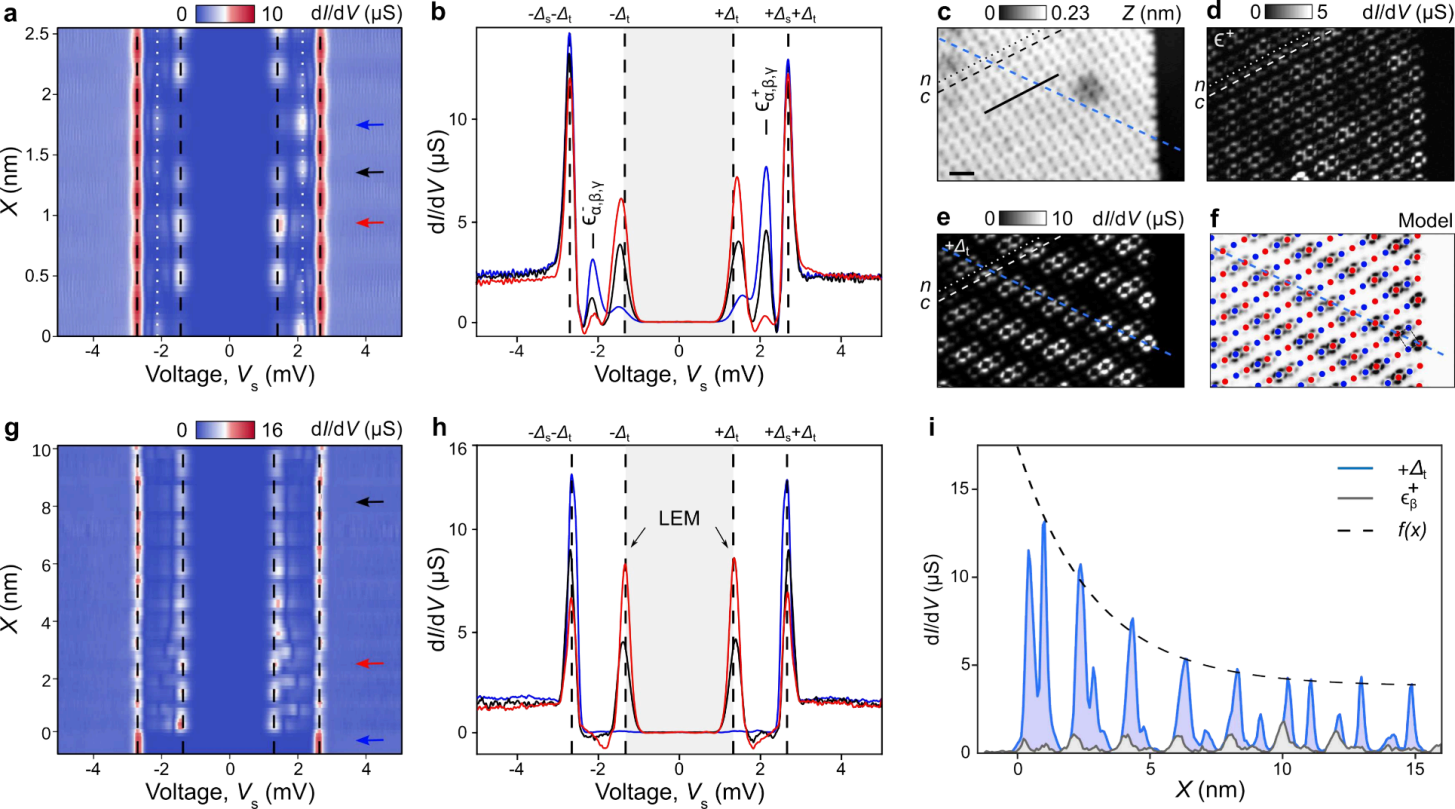
Tunneling spectroscopy of the LEM near an island edge using superconducting tips. (a) d*I*/d*V* cross-section acquired along seven molecules of a *c* row (black line in (c)). Lock-in parameters: *f* = 611 Hz, *A*_mod_ = 25 μV, tunneling parameters: *I*_*t*_ = 300 pA, *V*_s_ = 5 mV. White dotted lines are the ε_α,β,γ_^±^ energies. Dashed lines correspond to ±(Δ_t_ + Δ_s_) and ±Δ_t_, respectively. (b) d*I*/d*V* spectra extracted at positions marked by red, blue, and black arrows in panel (a). (c) STM topographic image of a supramolecular island edge. (d, e), Corresponding constant-height d*I*/d*V* maps recorded at +Δ_t_ (LEM energy) and at ε_α,β,γ_^+^ (YSR energies), respectively. Scale bar: 1 nm. (f) Schematic illustration of the spin structure (red: spin up; blue: spin down) superimposed on the LEM spatial distribution. The LEM lines are rotated by 60° with respect to the Pb(111) [110] direction. (g) d*I*/d*V* cross-section across the island edge along a single LEM line (blue dashed line in (c)). (h) d*I*/d*V* spectra at the blue, red and black arrows of (g). The LEM appears as a pair of peaks of equal magnitude at ±Δ_t_. (i) Spatial decay profile of the LEM wave function (blue) compared to that of YSR states (gray) along the same line cut in (c). The edge of the molecular island is set to *X* = 0 nm. The LEM envelope is tentatively fitted with two exponential components, yielding a short localization length ξ_1_ ≈ 3 nm and a long localization length ξ_2_ ≈ 80 nm.

Figure 3g shows a d*I*/d*V*(*V*,*X*) cross-section acquired along one LEM line marked by a blue dashed line in Figure 3e. All subgap excitations now appear at ±Δ_t_ with equal amplitudes between electron-like and hole-like regions (Figure 3h) in stark contrast with the intrinsic electron–hole asymmetry of YSR resonances (Figure 3b), which suggests a near zero-energy character. Note also that we are aware that the tip force can shift YSR states to zero-energy during a quantum phase transition (QPT) by influencing the magnetic exchange interaction between the impurity and the substrate.^25^ Such QPT experiment has been performed on an individual TBTAP^•–^ radical on Pb(111).^20^ It allows us to confirm that all d*I*/d*V* measurements presented here are obtained in the weak coupling regime, meaning that no relevant shift in energy of the YSR states and low-energy modes is expected by the influence of the tip. Tunneling experiments in the strong coupling regime will be discussed in a future work. To enable a better comparison with the mK measurements using metallic tips, we also deconvolute a characteristic d*I*/d*V* spectra of the near zero-energy states and display it in Figure S11. This results in an LDOS for a clean Pb surface which has symmetric coherence peaks at ±1.35 meV validating the deconvolution procedure. The deconvoluted d*I*/d*V* spectra also shows the edge state as single peak centered to zero-energy.

We next attempt to characterize the localization of the near zero-energy mode along one LEM line (Figure 3i) by comparing d*I*/d*V*(*X*) profiles at Δ_t_ with those recorded at the YSR energy ε^+^ (gray curve; also see Figures S15–S17). Unlike the continuous DOS associated with the YSR states, the wave function at Δ_t_ exhibits a pronounced maximum at the border of the island (*X* = 0) and decays toward the interior, without completely vanishing. In Figure 3i, the spatial profile of this edge-localized state is well captured by a two-component exponential decay (dashed line) yielding a short decay length ξ_1_ ≈ 3 nm and a much longer one ξ_2_ = 80 nm, as indicated by the dashed envelope fit in Figure 3i. As we will discuss below, the long localization length ξ_2_ is governed by the small topological gap, which is related to the value of the superconducting gap, whereas the short localization length ξ_1_ arises from significantly larger gaps induced by underlying magnetic ordering.

To support our findings, we used a tight binding model on a rectangular spin lattice following ref 33, which describes a spatial-symmetry-protected topological order of an antiferromagnet–superconductor hybrid (see the Supporting Information). The magnetic ordering of the spin lattice (schematized by red and blue arrows in Figure 4a) is deduced by comparing tunneling measurements on an individual TBTAP^•–^ with a dimer constructed by lateral tip manipulation (Figure S10). In comparison to the single molecule having one pair of YSR states at ε = ±100 μeV (Figure S10a),^20^ anionic molecules in a dimer configuration identical to that of *c* rows show their YSR states shifted to ε = ±320 μeV (Figure S10b) without splitting. The transition to a higher YSR energy for the dimer case is consistent with theoretical predictions of antiferromagnetic coupling of classical spins^51^ as well as previous experimental observations on FePc/NbSe_2_,^49^ whereas ferromagnetic order is expected to split the YSR states of each magnetic impurity. Given that the same trend is observed for the TBTAP^•–^ molecules in the assembly, we believe that an antiferromagnetic order is the most plausible scenario. Future studies using X-ray magnetic circular dichroism (XMCD) or spin-polarized scanning tunneling microscopy (SP-STM)^52^ should help to elucidate this point by directly probing the magnetic order in the molecular network assemblies.

**Figure 4:**
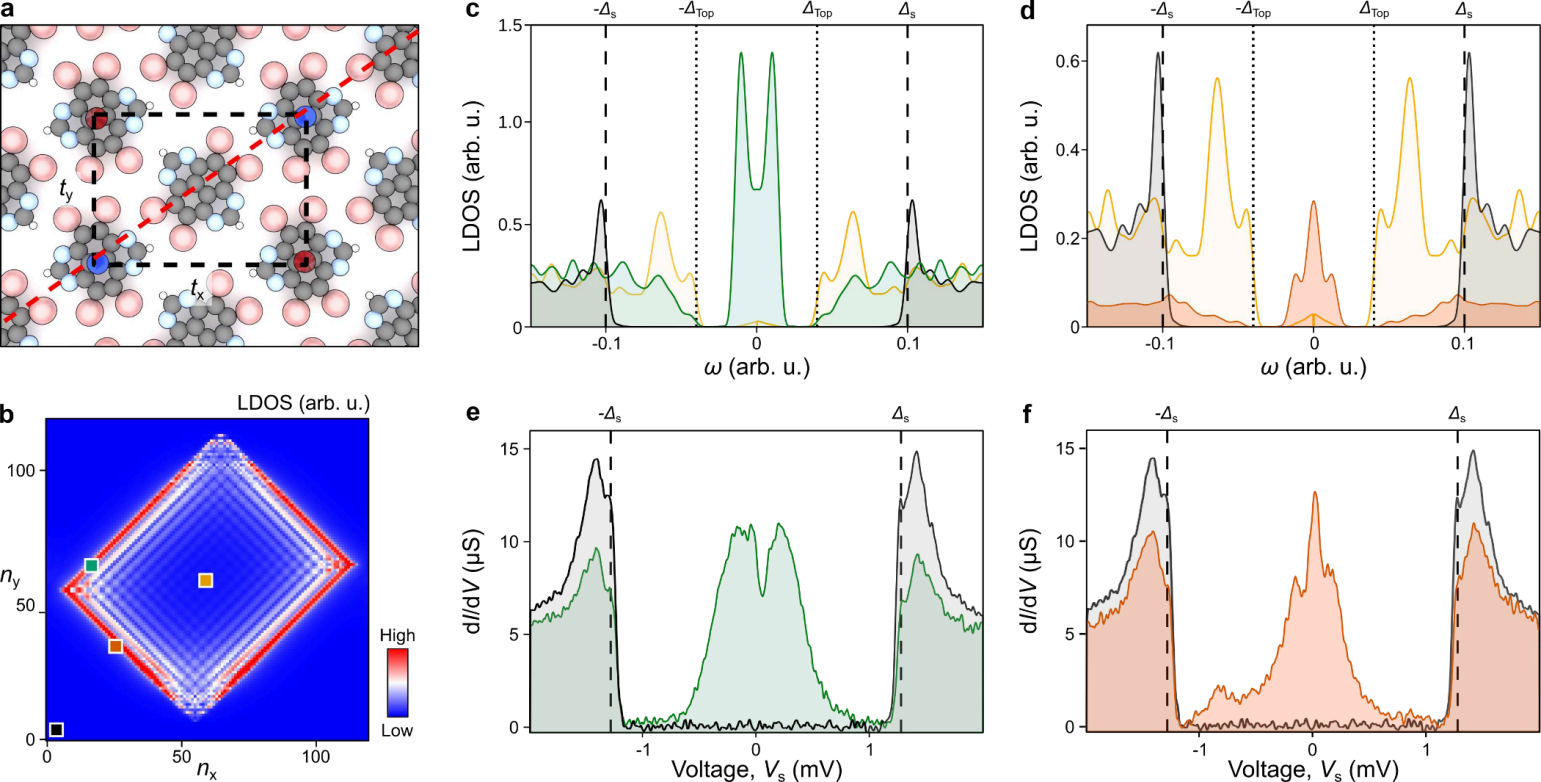
Comparison of LEM theoretical modeling with experimental data. (a) Antiferromagnetic spin lattice (dashed) formed by the supramolecular assembly with alternating spin orientations (red: spin up; blue: spin down). The underlying molecular lattice introduces anisotropic hopping parameters *t*_*x*_ and *t*_*y*_ along the *x* and *y* directions, respectively. The red dashed line indicates a 45°-rotated edge relative to the molecular lattice. (b) Calculated spatial map of the LDOS at zero energy, showing the emergence of zero-energy edge modes. (c, d) Theoretical LDOS­(ω) spectra of the LEM extracted at selected positions: a corner (green) and an edge (orange) of the island (as marked in (b)). The yellow spectrum corresponds to the island center, while the black spectrum is for the pristine Pb(111) substrate. Dashed lines indicate the superconducting gap edges (±Δ_s_); dotted lines denote the topological gap (±Δ_Top_). (e, f) Experimental d*I*/d*V* spectra of the edge modes acquired at 50 mK with a metallic tip. Lock-in parameters: *f* = 3.28 kHz, *A*_mod_ = 10 μV. Tunneling set point: *I*_t_ = 200 pA,*V*_s_ = 5 mV.).

The theoretical “Shiba” lattice was constructed to match the geometry by considering ferromagnetic edges aligned along the [1 10] directions with respect to the molecular lattice (red line in Figure 4a). The calculated LDOS at zero energy (Figure 4b) reveals low-energy modes (LEMs) consistent with edge states.^33^ Theoretical LDOS spectra are plotted in Figure 4c,d for two edge positions shown by colored squares in Figure 4b. They correspond to the vicinity of an island corner (green) and the middle of an edge (orange). The topological gap ±Δ_Top_ is defined in the island center (yellow), while ±Δ_s_ marks the superconducting gap outside the island (black). The LEM have two distinct spectral features consisting of either two peaks of equal amplitude split from zero (green in Figure 4c) or three resonances centered to zero energy (orange in Figure 4d). These signatures can be attributed to the strong anisotropy of the system, leading to two perpendicular edge states with distinct behaviors, and the two localization lengths (short and long) of the edge states. For example, near corners or defects, LEMs may exhibit additional side peaks due to these spatial decays. For comparison, Figure 4e,f (extracted at the locations marked by colored dots in Figures S17a,b) shows experimental d*I*/d*V* spectra acquired at *T* = 50 mK with a metallic tip near such an island corner, whose spectral signatures show strong resemblance to the theoretical LDOS of Figure 4c,d, respectively. Based on the data set in Figure 4b, the short localization length is estimated to be approximately three atomic sites, while the long localizations are of the order of the island size. Consequently, the LDOS spectra extracted at the center of the island (shown in yellow in Figure 4c,d) still exhibit a certain density of states at zero-energy due to the long localization lengths of the LEM.

Importantly, the 45°-edges of the antiferromagnetic island respect the underlying spatial symmetries, namely mirror symmetries,^33^ such that a gapped topological crystalline superconducting phase with topological edge states can form. The theoretical LEM have a short decay of few lattice sites (red area in Figure 4b) and a long localization length of the order of island size. The long localization length induces a small peak centered to zero-energy in the LDOS spectra extracted at the middle of the island (yellow spectra of Figure 4c,d). However, the tight-binding model does not reproduce the periodic modulation of the LEM observed in the experimental d*I*/d*V* map of Figure 3e. This discrepancy arises from the omission of the substrate in the simulations, which prevents the model from capturing the charge modulation induced by the commensurate alignment of the TBTAP domains on Pb(111) (cf. Figure 1e). Furthermore, the experimentally extracted long localization length, ξ_2_, varies significantly between different molecular domains. As discussed in Figure S12, this variation may be attributed to the influence of disorder and irregular island boundaries, which can break the required mirror symmetry and alter both the spatial profile and spectral features of the LEMs. We therefore propose that the large spread in ξ_2_ values stems from the complex and imperfect shapes of the molecular islands probed experimentally, which typically include more corners and defect sites than the idealized geometry considered in the theoretical model.

In conclusion, we demonstrate the formation of a two-dimensional electron spin lattice on superconducting Pb(111) via the supramolecular assembly of organic radicals. Each molecule in the *c* rows hosts a spin-1/2 state, confirmed by YSR in-gap states in tunneling spectroscopy. Low-energy edge states with long decay lengths and particle-hole symmetry were observed, consistent with a topologically nontrivial antiferromagnet–superconductor hybrid. Such a spatial-symmetry-protected topological superconductor has a complex phase diagram which crucially depends on the edge terminations of the system boundaries as well as the lattice parameter *a* (i.e., hopping parameters *t*),^33^ suggesting that tuning lattice parameters through chemical design could provide access to a range of phases.^53^ Additionally, creating local charge defects via probe chemistry^54^ offers a path to explore disorder effects on these edge modes. We also demonstrate reversible charge and spin control of individual radicals via local electric fields, enabling fine-tuning of the system. Integrating such molecular lattices into van der Waals heterostructures with back-gate control could unlock correlated phases, unconventional superconductivity,^55^ or Wigner crystallization.^56^ Our results point toward the potential of gate-tunable organic spin lattices on superconductors as possible platforms for exploring topological superconductivity.

## Supplementary Material



## Data Availability

The data underlying this article are available in Zenodo at 10.5281/zenodo.10613470.

## Notes

Materials and methods; details on the TBTAP assembly relaxed by DFT; d*I*/d*V* spectroscopy of anionic TBTAP^•–^ molecules; discharge of radical molecules by tip gating; details on the formation of “Coulomb” rings in d*I*/d*V* maps; Kondo resonance of TBTAP^•–^ under an external *B* field; antiferromagnetic exchange coupling between molecules; tight-binding model of irregular shaped islands; deconvolution of tunneling spectra with superconducting tips (PDF)
