# Supplementary material for: Toward Gate-Tunable Topological Superconductivity in a Supramolecular Electron Spin Lattice
Source: Nano Lett. 2025 Oct 8;25(42):15206–14. doi: 10.1021/acs.nanolett.5c03396 (PMC12550833; doi:10.1021/acs.nanolett.5c03396)
Supplement: Supplementary file 1 [file nl5c03396_si_001.pdf]

Supporting Information for : "Towards Gate-Tunable Topological Superconductivity in a Supramolecular Electron Spin Lattice"

Rémy Pawlak,[*,†] Jung-Ching Liu,[†] Chao Li,[†] Richard Hess,[†] Hongyan Chen,[‡] Carl Drechsel,[†] Ping Zhou,[¶] Xinyi Liu,[¶] Robert Häner,[¶] Ulrich Aschauer,[§] Thilo Glatzel,[†] Silvio Decurtins,[¶] Daniel Loss,[†] Jelena Klinovaja,[†] Shi-Xia Liu,[*,¶] Wulf Wulfhekel,[‡] and Ernst Meyer[†]

†*Department of Physics, WSS Research Center for Molecular Quantum Systems, University of Basel, Klingelbergstrasse 82, 4056 Basel, Switzerland*

‡*Physikalisches Institut, Karlsruhe Institute of Technology, Wolfgang-Gaede-Str. 1, 76131 Karlsruhe, Germany*

¶*Department of Chemistry, Biochemistry and Pharmaceutical Sciences, W. Inäbnit Laboratory for Molecular Quantum Materials and WSS Research Center for Molecular Quantum Systems, University of Bern, Freiestrasse 3, 3012 Bern, Switzerland*

§*Department of Chemistry and Physics of Materials, University of Salzburg, Jakob-Haringer-Strasse 2A, 5020 Salzburg, Austria*

E-mail: remy.pawlak@unibas.ch; shi.xia.liu@unibe.ch

Materials and Methods

Sample preparation

A Pb(111) sample purchased from Mateck GbmH was cleaned by cycles of Ar^+ sputtering and annealing using a radiative heater in ultra high vacuum (UHV). The 4,5,9,10-tetrabromo-1,3,6,8-tetraazapyrene (TBTAP) molecules were sublimed from a quartz crucible heated up to about 180 °C. The sublimation rate was measured with the quartz micro-balance. During evaporation, the Pb(111) sample was kept at $\approx$ 200 K and then immediately transferred to the low-temperature microscope.

High-resolution AFM imaging

The AFM experiments were carried out at T = 4.8 K using a low-temperature STM/AFM microscope from Omicron GmbH in UHV (p $\approx 1 \times 10^{-10}$ mbar), operated with a Nanonis RC5e electronics. The oscillator is a tuning fork sensor in the qPlus design[1] used in the frequency-modulation mode (resonance frequency $f_0 \approx$ 25 kHz, spring constant $k \approx$ 1800 N/m, quality factor $Q \approx$ 14000, and oscillation amplitude $A \approx$ 0.5 Å). The tip consists of a 25 µm-thick PtIr wire, sharpened by focused ion beam. A sharp tip apex was then prepared at low temperature by repeated indentations into the surface. Prior to AFM imaging, STM images at 4.5 K were acquired in constant-current mode with the bias voltage applied to the tip. To enhance the AFM contrast, we first tried to terminate the AFM apex with a single CO molecule preadsorbed on Pb(111). According to our experience, CO-tip functionalization is much more tedious on the Pb(111) substrate than on conventional Cu, Ag or Au metals.[2] Thus we use a single bromide atom as tip termination. For that, bromide atoms were dissociated from a single TBTAP molecule using voltage pulse of about 2,5 to 3,5 V. We then picked it up to the AFM apex by gentle indentation.

Tunneling spectroscopy at $T = 1$ K

Differential conductance spectroscopy dI/dV(V) spectra and maps were acquired with superconducting tips at the University of Basel with a custom-made Joule-Thomson STM/AFM microscope commercialized from Omicron GmbH. The microscope equipped with a 2.2 T dry magnet operates at 1.0 K with a SPECS-Nanonis RC5e electronics. STM tips were made from a 250 µm-thick Pb wire, which were sputtered for 2-3 hours in UHV to remove its native oxide. A superconducting tip was then prepared by repeated indentations into a clean Pb surface until a good superconducting gap is obtained. dI/dV spectra are acquired with the lock-in amplifier technique using a modulation of 610 Hz and a modulation amplitude of 15-50 µeV.

Tunneling spectroscopy at millikelvin temperatures

Superconductivity was investigated at millikelvin temperatures using the dilution refrigerated STM built in Karlsruhe Institute of Technology.[3] dI/dV spectra were recorded with the lock-in amplifier with the modulation frequency 3.2 kHz, and the modulation amplitude of tens of µeV (noted in captions).

Density-functional theory (DFT) calculations

Gas-phase DFT calculations were performed with the Gaussian 16 package,[4] using the B3LYP exchange-correlation functional[5] and a 6-31G** basis set. For the molecular anion, an extra electron was added and a doublet multiplicity initialized. DFT calculations for Pb(111)-adsorbed molecules were performed with the Quickstep code within the CP2K package,[6] employing the revPBE exchange-correlation functional[7,8] with a van der Waals (vdW) correction[9] and a mixed DZVP-MOLOPT-SR[10] Gaussian and plane-wave basis set,[11] the latter with a cutoff of 500 Ry. Electron-core interactions were described by Goedecker, Teter, and Hutter (GTH) pseudo-potentials.[12] The Pb(111) slab was modeled as a rectangular (5,

0)×(-2, 4) in-plane supercell of the primitive surface cell with dimensions 17.50 Å ×12.12 Å. The slab was four layers thick and a vacuum of 15 Å separates periodic images along the surface normal. In addition a 6×6 supercell of the primitive surface unit-cell was used for isolated molecules. In all cases, reciprocal space was sampled using the Γ-point only. Structural relaxations were performed until forces converged below 0.02 eV/Å.

Tight binding model

We utilize a tight binding model on a square lattice, similar to the one used in reference *(33)*, to describe spatial symmetry protected topological order in an antiferromagnet superconductor hybrid structure.

The kinetic part of the Hamiltonian is described by:

$$H_{\mathrm{kin}} = \sum_{n,m,\nu} \Big\{ -\mu_{n,m} c^\dagger_{n,m,\nu} c_{n,m,\nu} \\ \Big[-t_x c^\dagger_{n+1,m,\nu} c_{n,m,\nu} - t_y c^\dagger_{n,m+1,\nu} c_{n,m,\nu} + \mathrm{H.c.} \Big] \Big\} , \tag{1}$$

where, t_x $[t_y]$ and $\mu_{n,m}$ denote the hopping in x-direction [y-direction] and the chemical potential at the lattice site (n,m), respectively. Here, n $[m]$ denotes the x-coordinate [y-coordinate] of the lattice site and the total lattice size is given by $N_x \times N_y$. Moreover, the operator $c^\dagger_{n,m,\nu}$ $[c_{n,m,\nu}]$ creates [annihilates] an electron at site (n,m) with spin $\nu \in \{\uparrow, \downarrow\}$. We account for superconductivity via the term

$$H_{\mathrm{sc}} = \sum_{n,m} \left(\Delta c^\dagger_{n,m\uparrow} c^\dagger_{n,m\downarrow} + \Delta^*_n c_{n,m\downarrow} c_{n,m\uparrow} \right) , \tag{2}$$

where Δ denotes the superconducting gap. A Rashba spin orbit interaction is modeled via :

$$H_{\text{so}} = \sum_{n,m} \Bigg[\alpha_x \left(c^\dagger_{\downarrow,n-1,m} c_{\uparrow,n,m} - c^\dagger_{\downarrow,n+1,m} c_{\uparrow,n,m} \right) + i\alpha_y \left(c^\dagger_{\downarrow,n,m-1} c_{\uparrow,n,m} - c^\dagger_{\downarrow,n,m+1} c_{\uparrow,n,m} \right) + \text{H.c.} \Bigg], \tag{3}$$

where α_x [α_y] denotes the SOI strength in x-direction [y-direction]. Moreover, we choose the parametrization :

$$\alpha_x = \alpha \cos(\varphi) \qquad t_x = t \cos(\varphi) \tag{4}$$

$$\alpha_y = \alpha \sin(\varphi) \qquad t_y = t \sin(\varphi), \tag{5}$$

where the angle φ determines the ratio between the spin flipping (and normal) hopping terms in x and y-direction, while α and t set the maximal amplitude of the respective hopping terms.

Last, the exchange coupling is captured via the term :

$$H_J = \sum_{\substack{(n,m)\in\{\Lambda\},\\ \nu,\nu'}} (-1)^{n+m} J_{n,m} \left[\sigma_z\right]_{\nu,\nu'} c^\dagger_{n,m,\nu} c_{n,m,\nu'}, \tag{6}$$

where $J_{n,m}$ is the exchange coupling strength between the magnetic moments, which are antiferromagnetically aligned in z-direction, and the itinerant electrons. Furthermore, Λ is a subset of sites, which defines the size and shape of the magnetic island. This model supports spatial symmetry protected topological order. More precisely, a mirror symmetry combined with time reversal symmetry yields an effective time reversal symmetry. The combination of this effective symmetry with particle hole symmetry leads to a chiral symmetry enabling the definition of a topological order parameter, suggesting the presence of topological edge modes at boundaries which respect the underlying symmetry, see reference Ref. *(33)* for more details.

We solve the full Hamiltonian $H = H_{\text{kin}} + H_{\text{sc}} + H_{\text{so}} + H_J$ by exact diagonalization and find the eigenvalues E_η and the corresponding wave functions Ψ_η. The local density of states at the site (n, m) and at the energy ω can be calculated via :

$$\rho(\omega, n, m) = \sum_\eta \frac{1}{\pi} \frac{\kappa}{(E_\eta - \omega)^2 + \kappa^2} |\Psi_{\eta,n,m}|^2, \tag{7}$$

where κ accounts for temperature broadening. The tight-binding model was implemented via the python package Kwant.[13] We used the following parameters for Figure 4 presented in the main text: $t = 1$, $\Delta = 0.1$, $\varphi = 0.4$, $\alpha = 0.24$, $N_x = N_y = 120$, $\mu = 0.2 - 2t_x - 2t_y$, and $J = 2.4$.

Details on the TBTAP assembly relaxed by DFT

Figures S1A and B show the top and side views of the relaxed TBTAP assembly on Pb(111) obtained by density functional theory (DFT). The lattice parameter is $a_1 = 12.1$ Å $\times$ $a_2 = 17.5$ Å, rotated by $\theta = 90°$. In Figure S1A and B, TBTAP$^{\bullet -}$ molecules are displayed with their spin density (positive: yellow, negative: cyan). The STM image of Figure S1E resolves both the supramolecular assembly which the lattice is aligned along the $[1\bar{1}0]$ and $[1\bar{1}2]$ directions of the Pb(111). The experimental lattice parameters are in very good agreement with the relaxed structure of Figure S1A. From the side view of Figures S1B and C, we observe that all TBTAP molecules are flat lying in the same plane parallel to the Pb surface. However, STM/AFM images of the main manuscript show a difference of contrast from the two molecular charge-states coexisting in the self-assembly, which we recently reproduced by DFT in Reference 40.

Based on the DFT calculations of Reference 40, we simulate the AFM images using the Lennard-Jones force field and the Hartree potential electrostatics from DFT calculations considering the quadrupole model for CO-tip with various charge states. In Figure S1D, we show a series of simulated AFM images of the charged TBTAP assembly for positive ($q_{\text{tip}} =$

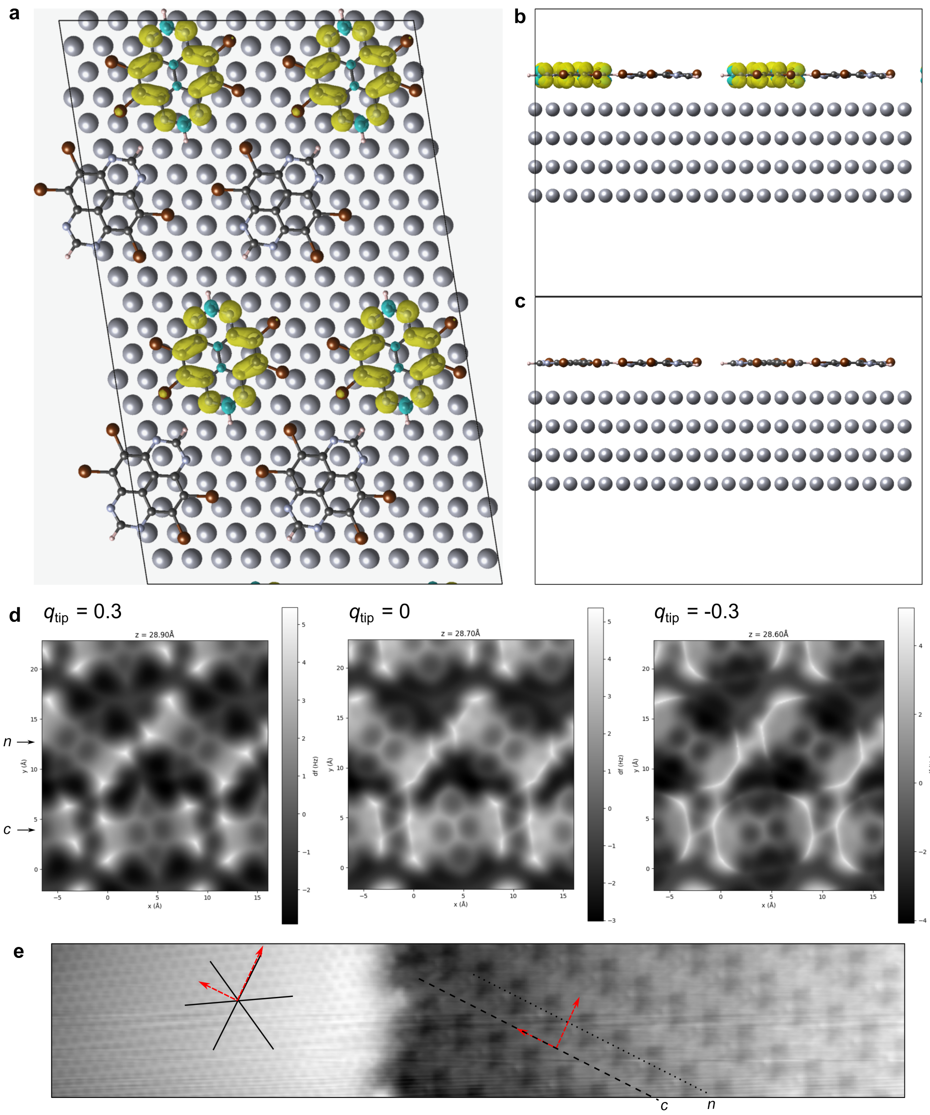

Figure S1: TBTAP supramolecular assembly on Pb(111) relaxed by DFT. (A) Top and (B) side views of the TBTAP supramolecular assembly on Pb(111). Black, blue, brown and gray colors refer to C, N, Br and Pb atoms. $TBTAP^{\bullet -}$ molecules are displayed with their spin density (positive: yellow, negative: cyan). (C) Side views of the TBTAP supramolecular assembly on Pb(111) without the spin density, showing the absence of structural differences between charged and neutral molecules. (D), Simulated AFM images with the probe-particle model using the Lennard-Jones force field and the Hartree potential electrostatics from DFT calculations, considering the quadrupole model for CO-tip for positive ($q_{\mathrm{tip}} = 0.3$), negative ($q_{\mathrm{tip}} =$ -0.3) and neutral ($q_{\mathrm{tip}} = 0$) charge-state of the tip. The arrows marked n and c points the neutral and charged rows, respectively. (E), STM image of both the supramolecular assembly and the Pb(111) substrate with atomic resolution. Red arrows refer to the molecular lattice along the $[1\bar{1}0]$ and $[1\bar{1}2]$ directions with respect to Pb(111) (black lines).

0.3), negative ($q_{\rm tip}$ = -0.3) and neutral ($q_{\rm tip} = 0$) charge-state of the tip. While a variation of the apparent bond lengths of the pyrene backbone can be observed between the simulations, we are unable to reproduce the overall increase of contrast between charged and neutral TBTAP molecules as observed in our experimental data of Figure 1e. We think that it may be related to the small magnitude of the Hartree potential and the absence of tip density of state in our simulation. This aspect of the contrast mechanism requires more investigations, which will address in future experiments.

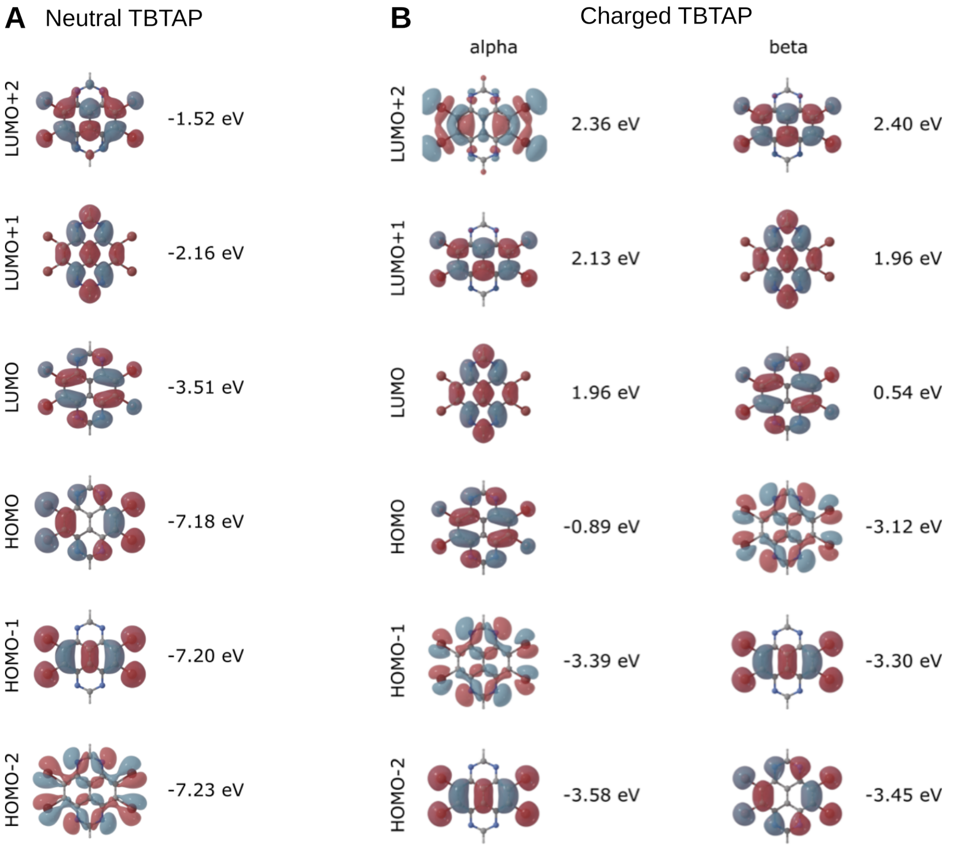

Figure S2: Molecular orbitals of neutral (A) and charged (B) TBTAP molecules.

dI/dV spectroscopy of anionic TBTAP molecules

Upon adsorption on Pb(111), the lowest unoccupied molecular orbital (LUMO) of the neutral TBTAP shifts below $E_{\rm F}$ and becomes singly-occupied by an electron donated by the surface.

As shown in Figure S3, this leads to a singly occupied molecular orbital (SOMO) of spin 1/2 which is accompanied by a singly unoccupied molecular orbitals (SUMO) above E_F due to Coulomb repulsion. Similar mechanism of the formation of radical molecules by electron donation from the surface have been reported in the literature.[14]

To estimate the energy position of the SOMO-SUMO levels, we acquired dI/dV spectroscopy on energies close to the Fermi level. Figure S4A shows dI/dV spectroscopic measurements of ten neighboring anionic molecules along a c row of the TBTAP assembly. Figure S4B is a single point-spectra of one of the molecules. i to iii marks the X position of three TBTAP$^{\bullet-}$ molecules of the row. Black arrows point to the SOMO-SUMO onsets at ± 150 meV, respectively. SC refers to the superconducting gap edge with its resolution hampered by the large voltage modulation (≈ 700 µeV) used for the lock-in detection. The dotted lines shows resonances symmetric to E_F outside the superconducting gap at ± 18 meV, ± 36 meV and ± 52 mV, which we attribute to vibrational excitations of the molecule.[14] These values of the vibrational excitations as well as the SOMO-SUMO levels are very close to those found for anionic TBTAP$^{\bullet-}$ adsorbed on Ag(111) (43).

Discharge of radical molecules by tip gating

The discharge of TBTAP$^{\bullet-}$ molecules on Pb(111) is obtained by applying a positive sample voltage V_s such as $V_s \geq V_D$. V_D refers to the threshold voltage at which a peak in the differential conductance is observed as a result of the molecule's discharge (see Fig. 1 of the main manuscript). As described in reference (35), this behavior is interpreted by a double barrier tunneling junction (DBTJ) schematized in Figure S5. Increasing the sample voltage V_s gradually shifts the energy level of the singly-occupied state of the molecule (i.e. SOMO) by the amount $\mathcal{L} \cdot V$ with respect to the chemical potential of the substrate. For $V_s = V_D$, the SOMO level crosses the surface chemical potential and becomes empty. The molecule is thus in a TBTAP^0 neutral state (see arrow in Figure S5B). This charge-state transition leads

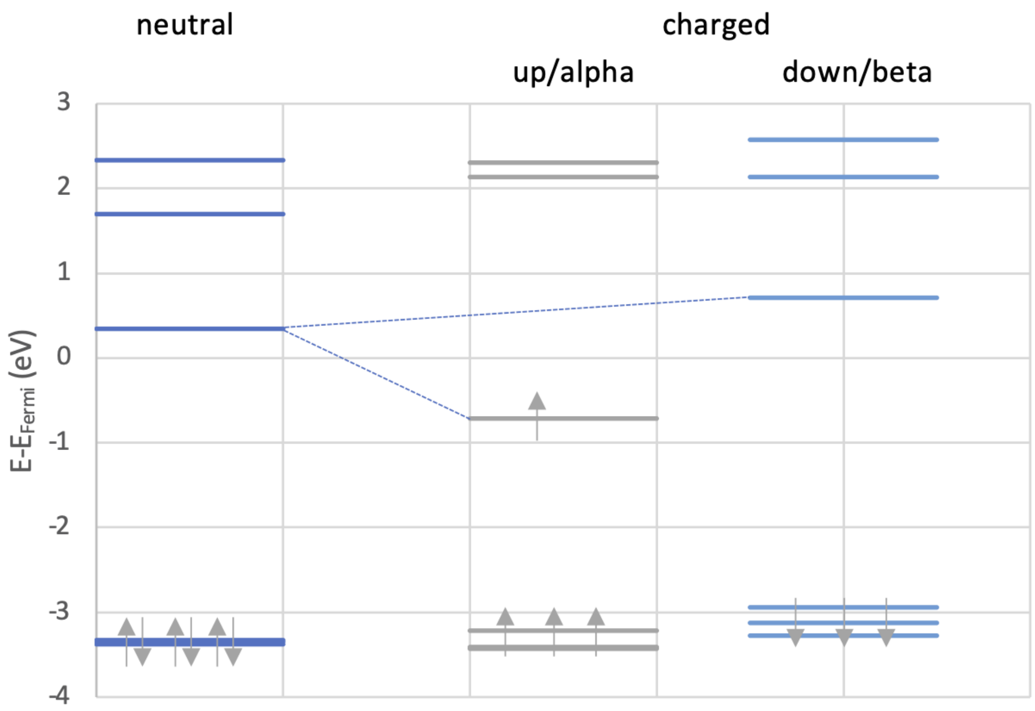

Figure S3: Comparison of the gas-phase energy levels between neutral and charged TBTAP molecule. Upon charging of the former LUMO level, the anionic $TBTAP^{\bullet-}$ molecule split into a singly occupied molecular orbital (SOMO) and a singly unoccupied molecular orbital (SUMO). The SOMO carries an electron spin.

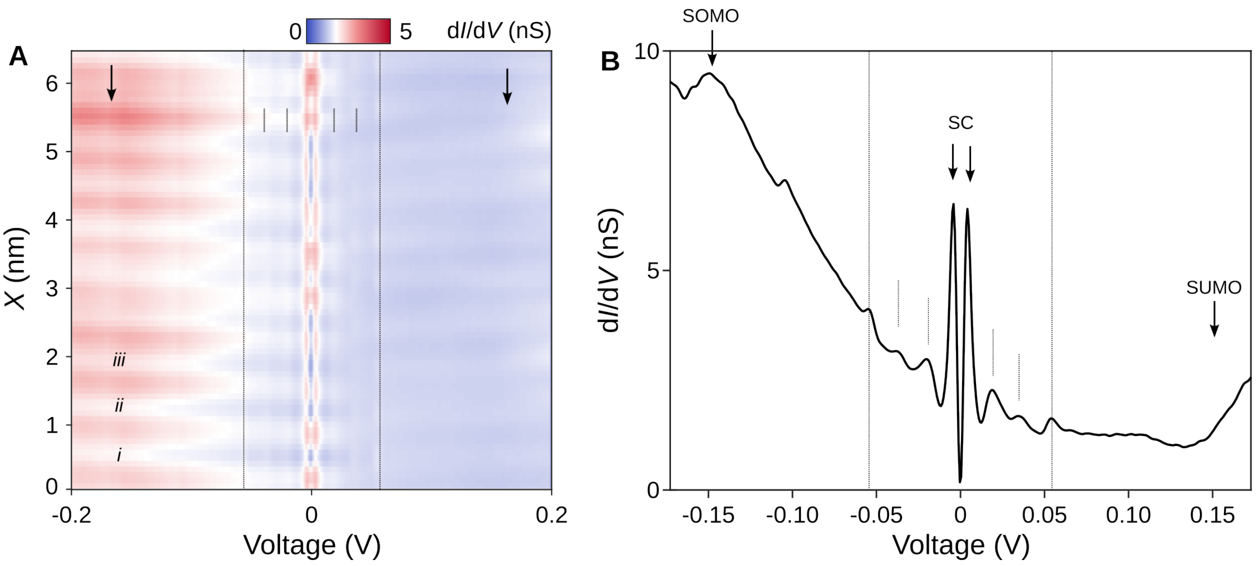

Figure S4: dI/dV spectroscopy of anionic TBTAP molecule near E_{F}. (A),dI/dV cross-section taken along ten molecules of a c row. (B), dI/dV point-spectra extracted at a molecule position. Dotted lines refers to vibrational excitations of the TBTAP molecule at $\pm$ 18 meV, $\pm$ 36 meV and $\pm$ 52 meV, respectively, which are similar to our previous work of TBTAP/Ag(111) (33) . i to iii marks the X position of three TBTAP$^{\bullet-}$ molecules of the row. Black arrows point to the SOMO-SUMO onsets at $\pm$ 150 meV, respectively. SC refers to the superconducting gap edge which resolution is hampered by the large voltage modulation used for the lock-in detection (Lock-in parameters: f = 611 Hz, A_{mod} = 700 µeV, tunneling parameters: I_{t} = 100 pA, V_{s} = 100 mV.).

to an increase of the tunneling current between tip and sample (arrow in Figure S5B), better observed in differential conductance as a sharp peak (Figure 1f of the main manuscript).

The energy shift ΔE of the SOMO directly amounts with the electric field and is proportional to the voltage V_s such as: $\Delta E = e\mathcal{L}V$, with e the elementary charge and $\mathcal{L}$ the lever arm. As described in Refs. (41-43), α simply expresses as the ratio between the SOMO level E_SOMO and the energy of the discharge $E^{-\to 0} = \mathrm{e}V_\mathrm{Thresh}$ such as $\mathcal{L} = E_\mathrm{SOMO}/(E^{-\to 0} - E_\mathrm{SOMO})$. We estimate E_SOMO = -150 meV (Figure S4) and $E^{-\to 0}$ = 0.9 eV (1 F of main manuscript) which results in $\mathcal{L}$ = 0.15. This value is in a similar range compared to previous works showing the discharge of molecules on metals (41) or on thin insulating layers.[15]

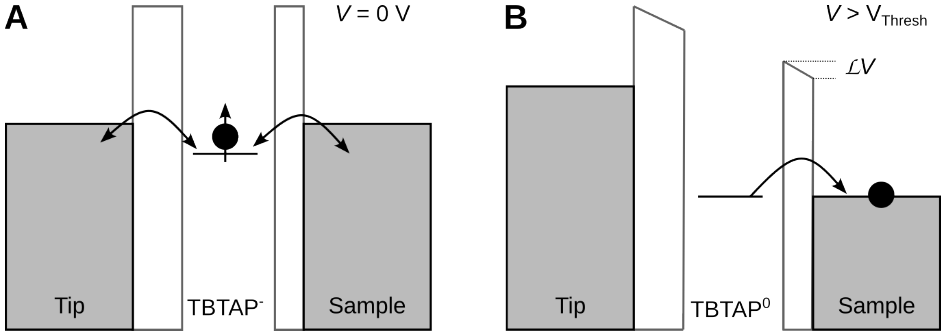

Figure S5: Discharge of anionic TBTAP by the electric field of the tip using the DBTJ model. (A), Energy levels of the tip-TBTAP-sample for V_s = 0 V. Tunneling barriers are in pale gray. The SUMO of the molecule below E_F is occupied by an electron. (B), By applying a positive voltage $V_\mathrm{s} \geq V_\mathrm{Thresh}$, the energy level of the SOMO shifts by the amount $\mathcal{L}V$ with respect to the chemical potential of the substrate. When the SOMO crosses E_F, the level is emptied leading to the discharge and the neutral state of the TBTAP molecule.

Details on the formation of "Coulomb" rings in dI/dV maps

The lever arm $\mathcal{L}$ represents the efficiency with which the STM tip can discharge the SOMO level of the anionic TBTAP$^{\bullet-}$ where a single electron is trapped. The discharge mechanism is described in Figure S5. The process is dictated by a capacitive coupling between tip and molecule, which implies that α linearly depends on the applied voltage V_s as well as the (X,

Y, Z) positions of the tip with respect to the molecule.

In the case of 0D quantum dot-like systems (as expected for the anionic TBTAP$^{\bullet-}$ molecule), a single discharging event for a constant tip-sample separation Z for $V_\mathrm{s} \geq V_\mathrm{D}$ appears in constant-height dI/dV map as a dot/ring of high differential conductance (Fig. 1 of the main manuscript). As V_s increases for a constant tip-sample separation Z above the singly-occupied molecule, the discharging fingerprint in dI/dV map evolves from a dot at $V_\mathrm{s} = V_\mathrm{D}$ (bottom of the parabola) to a ring ($V_\mathrm{s} \geq V_\mathrm{D}$) exemplary shown in red (top of the parabola). The ring diameters linearly increase with V_s as the electric field of the tip discharge more efficiently the molecule even at larger lateral (X, Y) distances. This is depicted by the series of concentric rings contained in the paraboloid in Figure S6A positioned on top of the punctual charge at the surface. Inside such a ring depicted in Figure S6B, the tip discharge the QD underneath (region e =1) while no charge is being removed when outside the rings ($e = 0$).

In the case of two non-interacting 0D-quantum dots (Figure S6B), each QD exhibits a similar series of voltage-dependent rings centered to their position. By considering that the left and right QD have the same discharge voltage V_D (i.e. same on-site Coulomb interaction U) but no hopping amplitude t between them, increasing V_s promotes the local discharge of each dot ($e = 1$) when the tip is in its vicinity during a scan. This leads to the formation of identical ring sizes ($V_\mathrm{s} = V_1$ in Figure S6D) located at the QD positions in dI/dV maps (Figure S6E). When $V_\mathrm{s} = V_2$ corresponding to the crossing of parabolas, a double discharge event ($e = 2$) is possible at (X, Y) positions equidistant from the two QDs. In that case, the tip field simultaneously couples to both left and right QDs as depicted in Figure S6F. In dI/dV maps, such double discharge manifests as the crossing of neighboring rings and their intersection as shown in Figure S6E.[16] Inside the area of the intersection, two charges are being removed by tip gating ($e = 2$, Figure S6F).

In the case of two coupled 0D-quantum dots with a hopping t between neighboring QDs (Figure S6G), each QD has the same series of rings centered to their position as for the non

interacting QD case. When $V_s \geq V_2$, neighboring rings fuse in dI/dV maps as shown in Fig. 2H of the main manuscript. The absence of intersection for ever increasing V_s in dI/dV maps indicates the double discharge of both QDs for all (X, Y) position inside the area $e = 2$ of Figure S6H. Therefore, the tip gating allows the discharge of both QDs even if the tip is not located directly in the vicinity of the QD (Figure S6I).

This important observation (i.e. ring fusion in Figure S7E) implies that each $TBTAP^{\bullet-}$ molecules carrying one electron, which are localized on the molecular sites of charged row c. By applying a sufficient gate voltage $V_s \geq V_2$, the system is locally doped with one hole (i.e. by removing one electron of the row) allowing electrons to flow between first neighboring sites. The charged row c thus become metallic at such tip gating. This transition between the insulator and metallic state is known as Mott transition.[17] It also allows us to conclude that the $TBTAP^{\bullet-}$ electrons are strongly correlated in the supramolecular assembly. Note also that we tried to further increase the gating voltage V_s in order to explore the electron correlation between neighboring c rows in dI/dV maps. Unfortunately, the applied voltage of about 1.5-2.0 V is close to the threshold value for the tip-induced debromination of the molecule. As a result, such experiment usually damages the supramolecular assemblies instead of the observation of the intersection/fusion of Coulomb rings between these rows.

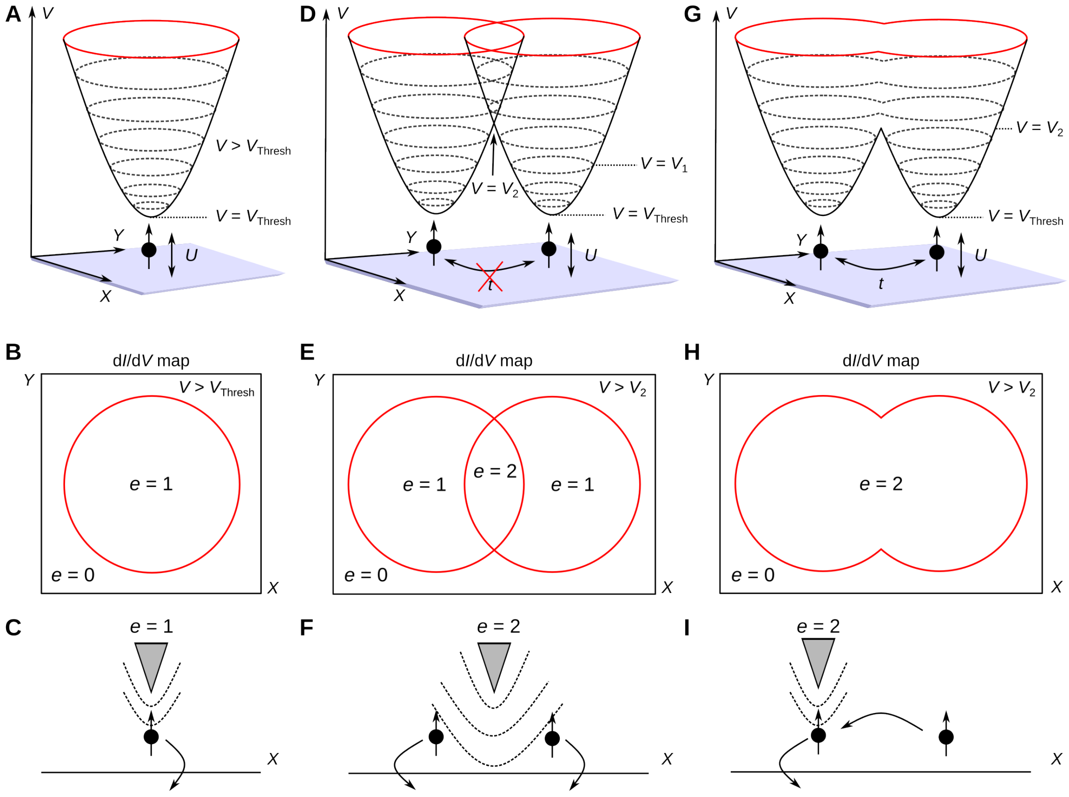

Figure S6: Formation of Coulomb rings in quantum dot-like systems. (A), A quantum dot system with on-site Coulomb interaction U can be discharged by the electric field of the tip when $V_{\mathrm{s}} \geq V_{\mathrm{Thresh}}$. (B), Upon scanning at constant tip-sample separation Z, the discharge event appears as dot/rings in dI/dV map, which diameters linearly depends on the applied voltage. Inside the ring, the tip efficiently couples with the QD provoking the discharge (e =1) as depicted in (C). (D), Two non-interacting quantum dots with on-site Coulomb interaction U can be similarly discharged by the electric field of the tip when $V_{\mathrm{s}} \geq V_{\mathrm{Thresh}}$. At $V_{\mathrm{s}} = V_2$, the paraboloids centered to each QD start crossing leading to the intersection of the Coulomb rings in dI/dV map (E). The area $e = 2$ corresponds to the (X, Y) positions of the tip allowing the discharge of both QDs as depicted in (F). (G), Two interacting quantum dots with on-site Coulomb interaction U and hopping integral t can be discharged by tip gating when $V_{\mathrm{s}} \geq V_{\mathrm{Thresh}}$. In contrast to the non-interacting case, applying $V_{\mathrm{s}} = V_2$ leads to the fusion of the Coulomb rings in dI/dV map (H). In the area e =2, the tip allows the discharge of both QDs even if the tip is not located directly above one QD. The ring fusion is the fingerprint of electron hoping between neighboring molecular sites (I).

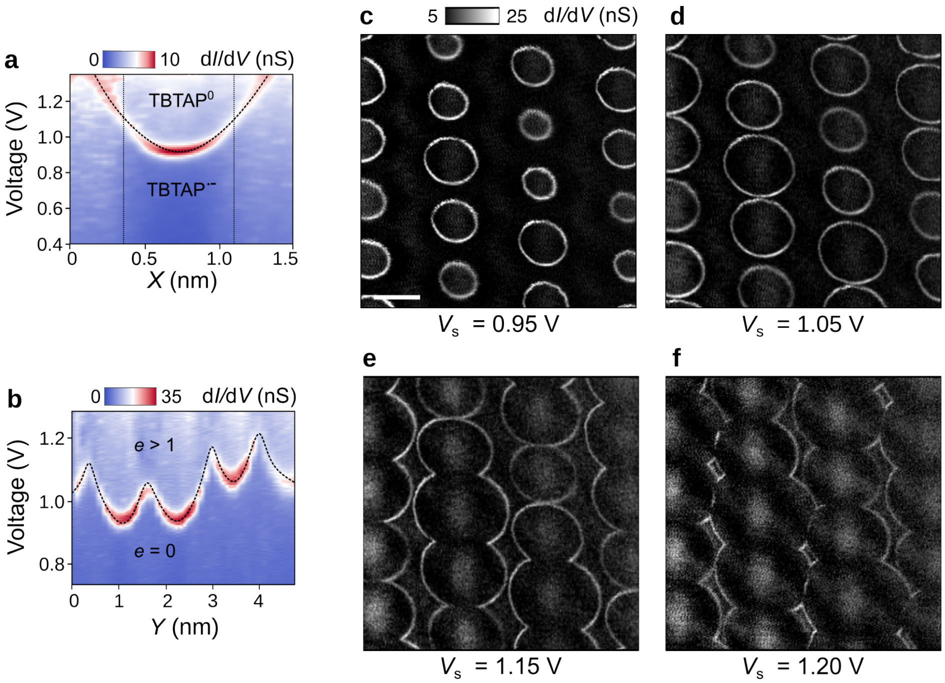

Figure S7: Discharge of anionic TBTAP by the electric field of the tip using the DBTJ model. (A), dI/dV cross-section across $n-c-n$ rows showing the electron localization to the charged molecule. (B), dI/dV cross-section across five TBTAP$^{\bullet -}$ molecules (dashed line in **a**). The dashed line corresponds to multiple discharge events (e is the number of removed charges) induced by tip gating. (C-F), Series of dI/dV mapping for increasing tip-sample voltage V_{S} showing the expansion of the ring diameter and cascade discharge for $V_{\mathrm{S}} \geq 1.15$ V. Scale bars are 1 nm.

Kondo resonance of TBTAP$^{\bullet-}$ under an external B field

To further confirm the magnetic nature of the anionic TBTAP$^{\bullet-}$, we quenched the superconductivity state of Pb(111) to normal state in order to probe the Kondo resonance. For that we applied an out-of-plane magnetic field of about 0.5 T and measured dI/dV point-spectra on charged and neutral molecules (Figure S8*A*). By performing temperature-dependent dI/dV measurements, the zero-bias peak broadens as T increases and almost vanishes for $T = 10.5$ K.

Under the external magnetic field of about 0.5 T, we also acquired grid spectroscopic measurements in the middle and at the border of the supramolecular assembly (Figure S11).

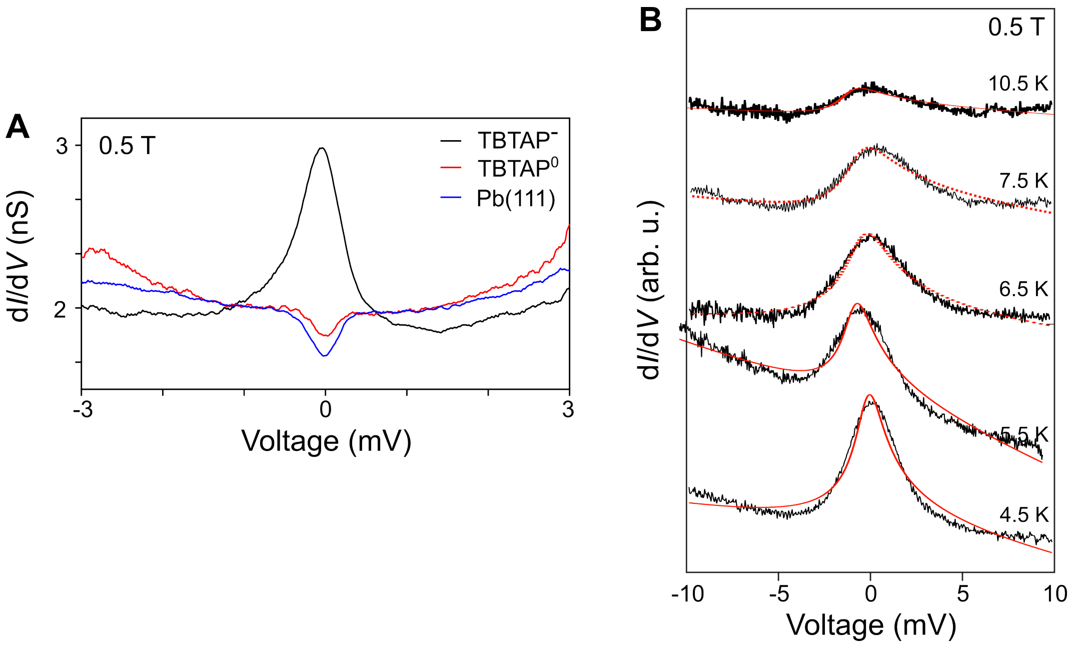

Figure S8: Kondo resonance of TBTAP$^{\bullet-}$ under an external B field. (A), dI/dV spectra acquired at the center of the molecule with a vertical B field of 0.5 T. On TBTAP$^{\bullet-}$ (black spectra), the zero-bias peak is attributed to a Kondo resonance from its electron spin. On neutral TBTAP (red), no signature of the Kondo resonance is observed but rather a dip at zero energy. Since this dip is also present on the pristine Pb(111) (blue spectra), we attribute it to the small superconducting gap of the tip which has not completely vanished at the B field of 0.5 T. The position of the spectra are shown with colored dots in Figure S9C. (B), Temperature-dependent dI/dV spectra of the TBTAP$^{\bullet-}$ molecule. Black spectra are the experimental data shifted for clarity, while red spectra are the fit using the Frota function.

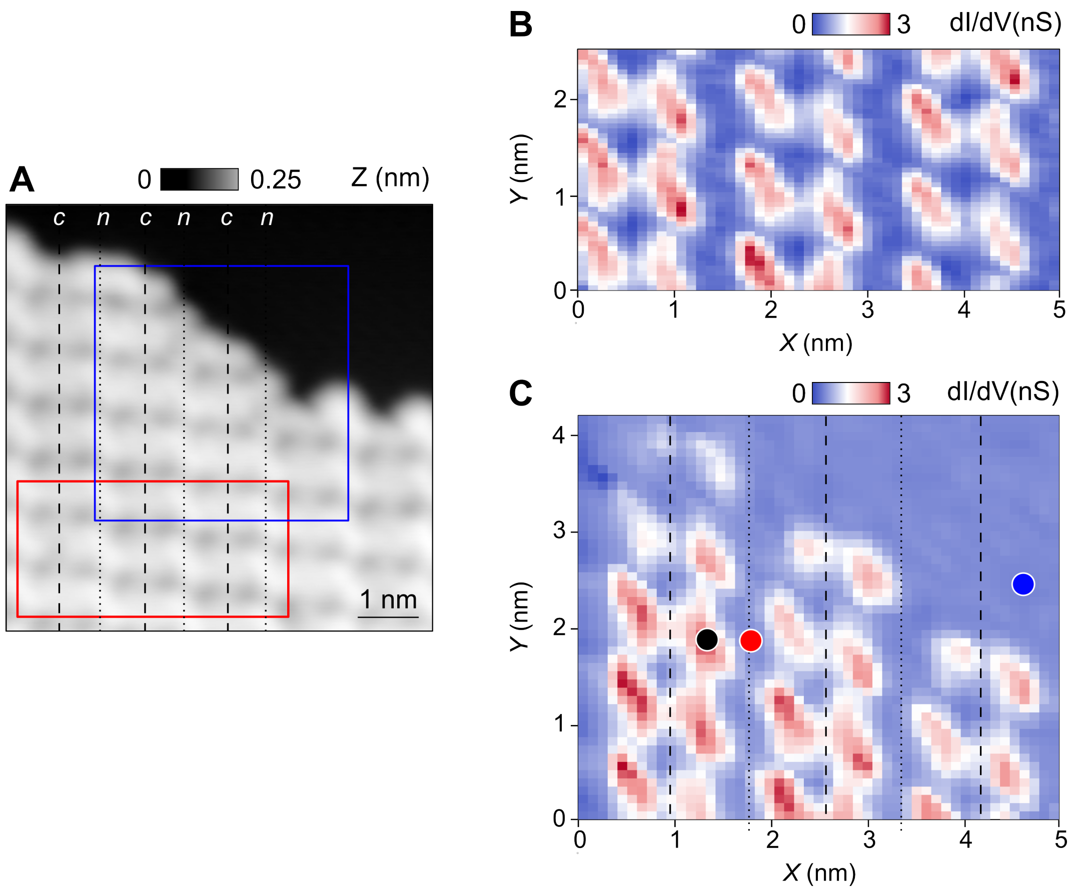

Figure S9: Zero-energy dI/dV grid spectroscopy under an external B field. (A), STM image of the TBTAP assembly on Pb(111) under an external B field of 0.5 T at at the temperature of 1 K. Red and blue rectangles corresponds to the position of two sets of grid spectroscopic measurements. (B), Zero-energy map extracted from the dI/dV grid spectroscopy marked in red. (C), Zero-energy map extracted from the dI/dV grid spectroscopy marked in blue. Rows labeled c and n correspond to charged and neutral molecules, respectively.

Antiferromagnetic exchange coupling between molecules

To probe the YSR states of the single impurity case, we extracted using tip manipulation single TBTAP$^{\bullet-}$ molecules from an island (Figure S10A). Despite the manipulation process, the molecule remains singly occupied on the Pb(111), which is confirmed by clear YSR subgap states in dI/dV spectra acquired at the center of the molecule (Figure S10). The YSR level of the single impurity case is $\varepsilon_b = \pm\ 100\ \mu$eV, which drastically differs with the ones reported in the main manuscript (i.e. $\varepsilon_\alpha^\pm$, $\varepsilon_\beta^\pm$ and $\varepsilon_\gamma^\pm$). The equation :

$$\varepsilon_b = \Delta \frac{1-\alpha^2}{1+\alpha^2}$$

allows us to to determine the parameter $\alpha = 0.92$, where $\Delta = 1.35$ meV is the superconductive gap. As seen in Figure S10D, the dI/dV spectrum of the molecular dimer shows a shift of the YSR states at each monomer to larger energies $\varepsilon_b = \pm 320\ \mu$eV. No splitting of the YSR-levels of a dimer is observed, indicating an antiferromagnetic coupling of the electron spins. The value of the shift can be compared with the equation :

$$\varepsilon_b = \Delta \sqrt{\frac{\left(1-\alpha^2\right)^2 + 2(\alpha/k_F r)^2 + d}{(1+\alpha^2)^2 + 2(\alpha/k_F r)^2 \cos 2k_F r + d}}$$

where

$$d = \alpha^4 \left(1 + 2\cos 2k_F r\right) \left(\frac{1}{k_F r}\right)^4$$

and $\Delta = 1.28$ meV is the superconductive gap, $\alpha = 0.92$, $k_F = 5.4$ nm^{-1} is the Fermi wave vector, r the separation between molecules (Eq. 9 from Reference *(33)*). At a separation $r =$ 1.0 nm, the energy ε is ± 0.2 meV, which is in reasonable agreement with the experimental value. Therefore, we can conclude that the spins located on the TPTAP-molecules are antiferromagnetically coupled. In the antiferromagnetic coupling case, it is possible for a pair of YSR-states to become virtually occupied by Cooper pairs from the superconductor,

which gives the resonant enhancement.[18]

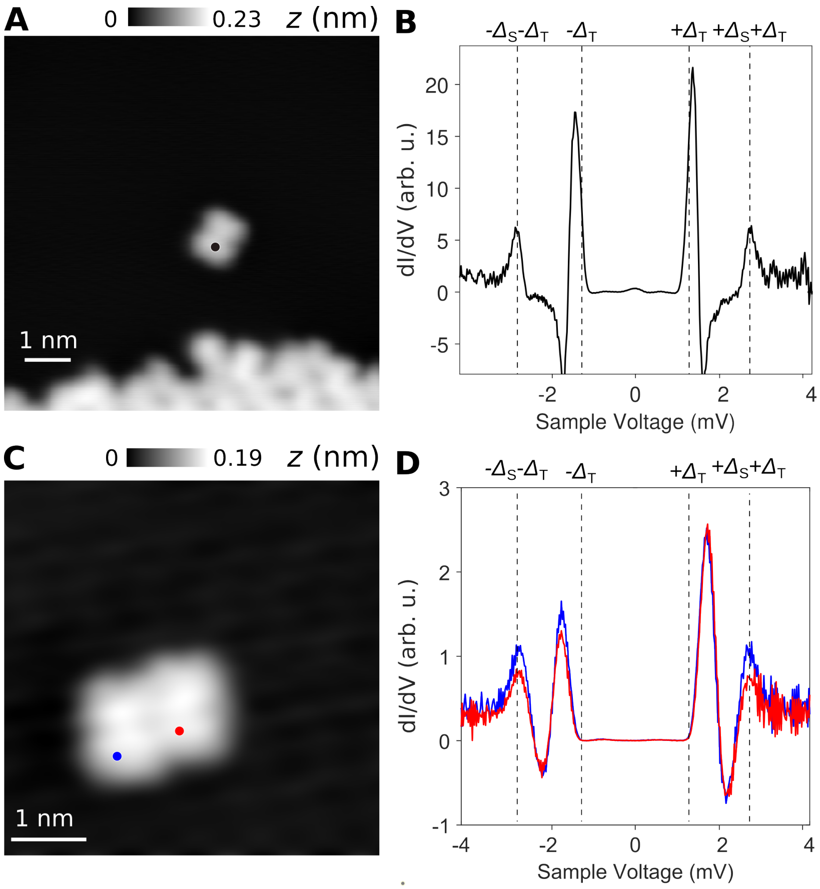

Figure S10: dI/dV measurements of the TBTAP$^{\bullet -}$ dimer. (A), STM image after the tip-induced manipulation of a single TBTAP molecule from the assembly. (B), dI/dV spectra of the single molecule acquired at 1 K at black dot in (A). Dashed lines lines correspond to $\pm$ ($\Delta_T + \Delta_S$) and $\pm$ Δ_T energies, respectively. The YSR resonances, at $\pm$ 145 µeV and $\pm$ 165 µeV, indicates that the molecule. Lock-in parameters: f = 611 Hz, A_{mod} = 20 µeV. (**C**), STM image after the formation of a dimer by tip manipulation. (**D**), dI/dV spectra of each molecule acquired at 1 K with a Pb tip.

Deconvolution of tunneling spectra with superconducting tips

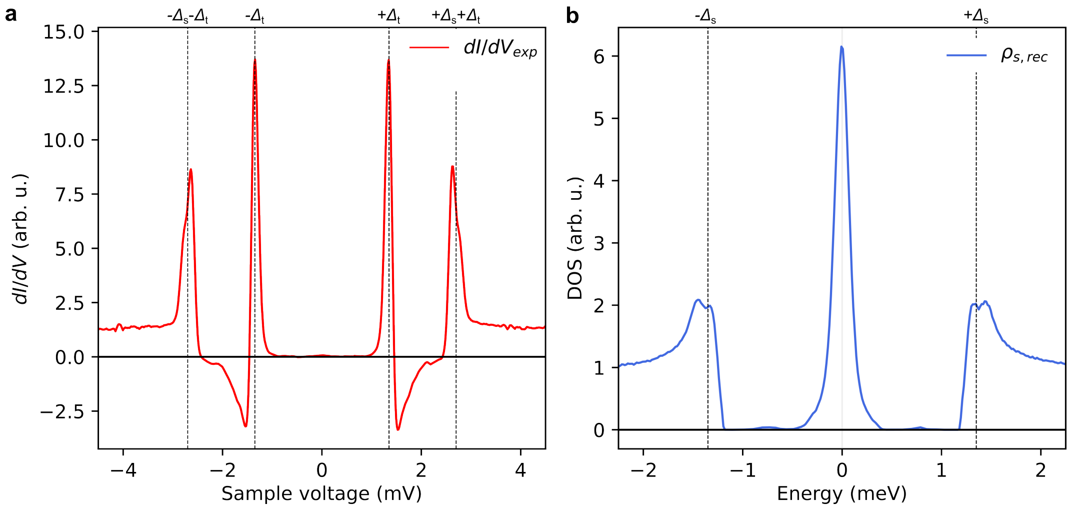

Figure S11: Deconvolution of $\mathrm{d}I/\mathrm{d}V$ spectra with superconducting tips. (A), Experimental $\mathrm{d}I/\mathrm{d}V$ spectra acquired at 1 K with a superconducting Pb tip showing the low-energy mode at the edge of a molecular island. Lock-in parameters: f = 610 Hz, A_{mod} = 15 µV, T = 1 K, tunneling parameters: I_{t} = 200 pA,V_{s} = 5 mV. (B), Deconvoluted spectra of the experimental data. The set of parameters for Pb(111) surface are $\Delta_{\mathrm{s}} = \Delta_{\mathrm{t}}$ = 1.35 meV, γ_{s} = 0.03 meV and A = 0.83 that corresponds to the ratio between the intensities of the two DOS contributions 5:1. The surface DOS also contains a contribution from the YSR states modeled as an additional symmetric pair of Loretzian peaks at varying energy ω_{YSR} , width γ_{YSR} = 0.02 meV and weight ω_{YSR} = 0.35. Calculations are performed at $k_{\mathrm{B}}T$ = 0.08 meV equivalent to about 1.0 K.

Tight-binding model of irregular shaped islands

In the main part of this paper, we analyzed a magnetic island whose boundaries form a 45°-angle with the underlying square lattice. Here, instead we study edge modes in an irregular shaped island. In fact, we design an island with five different types of boundaries: Four of them respect the reflection symmetry of the system while one boundary, shaped as a quarter of a circle, explicitly breaks the underlying mirror symmetries. We find that edge modes are well localized at an edge parallel to the y-axis and to boundaries rotated by 45° with respect to the lattice structure, see Figure S12A. In contrast, there is no sub-gap state localized at the boundary parallel to the x-direction, since the associated local topological invariant is zero, see also reference (33). The curved boundary does not fully suppress the existence of edge states: there is an edge mode running along a sizeable fraction of the irregular boundary. However, this mode is not topological protected. In general, we find a similar behavior as described in the main text: the local edge states lead to peaks in the LDOS at energies smaller than the proximity gap. These peaks can split into several peaks, see Fig. Figure S12B. The shape of these peaks is determined by the system parameters, the form of the corresponding boundary, and the ratio between the distance of the position to the next corner and the localization length.

Next, we analyze the effect of small disorder on the system. In particular, we add locally random potential terms from a Gaussian distribution to the chemical potential. The strength of the disorder is set by a standard deviation σ_μ. This type of disorder obviously breaks the reflection symmetries. Therefore, the concept of symmetry protected topological order does not apply to the system. However, for small disorder strength, meaning that $\sigma_\mu < \Delta$, we still find an accumulation of the DOS at the edges for sub-gap energies, see Figure S12C. We explain this phenomenon by the adiabatic behaviour of physical systems: a small change of the Hamiltonian parameters can not completely change the energy spectrum. We note, however, that in contrast to topological systems the original energies can split easily away from their initial values. We suggest an analogy to the SSH chain: The addition of

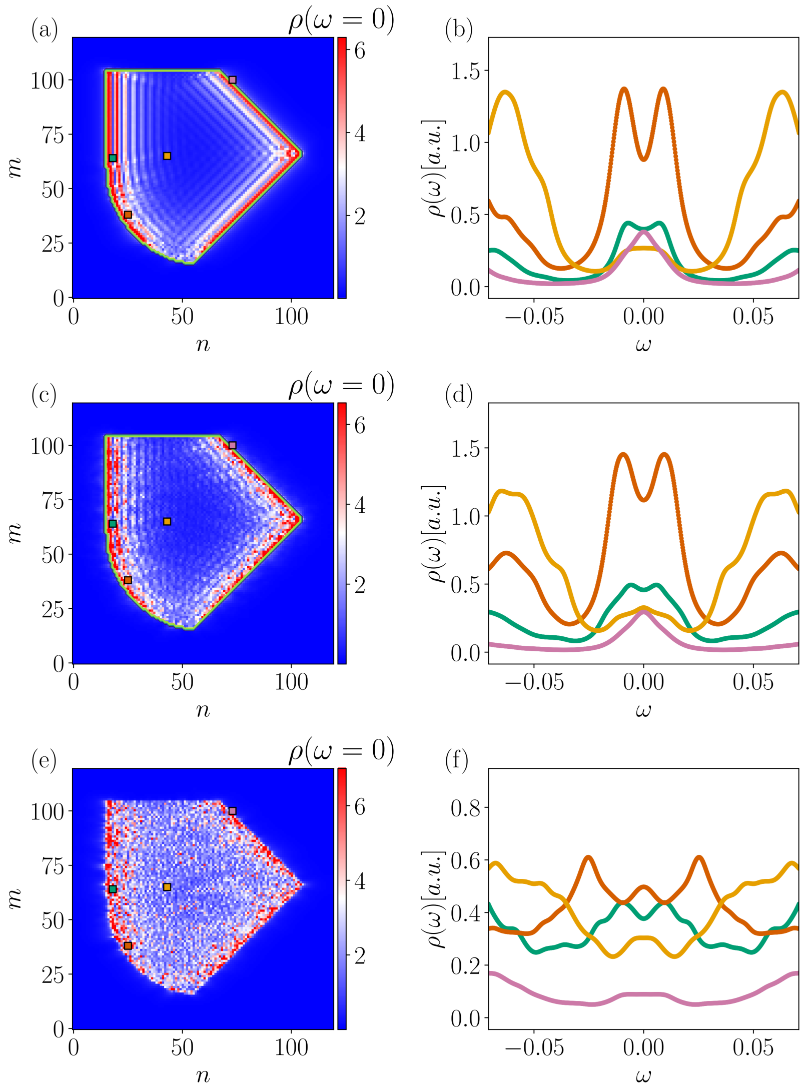

Figure S12: Edge states in an irregular shaped island. Left column: The local density of states at zero energy reveals localized states at certain types of boundaries; the boundary itself is indicated in (a,c) by a bright green line. Right column: Local density of states as a function of energy, evaluated at the positions of the same-colored markers in the respective panel (a,c,e). While the edge states are clearly visible for (a,b) zero disorder and (c,d) small potential disorder, which breaks the mirror symmetries, the states are almost fully removed in case of magnetic disorder of the same strength. $t = 1$, $\mu \approx 0.2 - 2t_x - 2t_y$, $\alpha = 0.24$, $\varphi = 0.4$, $\Delta = 0.1$, $\kappa = 0.005$, $N_x = 120$, $N_y = 120$, $J = 2.4$, (a) $(\sigma_\mu, \sigma_J) = (0, 0)$, (b) $(\sigma_\mu, \sigma_J) = (0.075, 0)$, (c) $(\sigma_\mu, \sigma_J) = (0, 0.075)$

random disorder to the nearest neighbor hopping does respect the chiral symmetry of the SSH chain. Consequently, the topological protection is preserved and the edge state is fixed to zero energy. In contrast, a perturbative modulation of the chemical potential breaks chiral symmetry and the edge state can deviate from zero energy. If this potential perturbation in the SSH chain is not too strong, than the original zero-energy state remains localized at the edge of the system and acquires a finite random energy inside the gap.

We observe here a similar behavior of the edge states in the antiferromagnet Superconductor heterostructure. They are not protected against the chosen disorder, and change their energy, however, for sufficient small disorder they remain at the former symmetry preserving boundaries of the system. Stronger perturbations easily remove the edge states and lead to localized states in the bulk of the island. Last we study disorder in the exchange coupling strength, described by a standard deviation σ_J. This type of disorder breaks the symmetry as well and we find that it has a stronger effect than potential disorder. In fact, we find for the same value of standard deviation that the edge states are almost removed from the system, see Figure S12E. In the experiment, STM scans show a low defect density in the lattice, therefore we assume that the model based on spatial symmetry protected topological order could explain the LEM. However, we emphasize that it is not possible to rule out alternative explanations of the LEM and that the model discussed here does not capture the quantum nature of the spin. Therefore, further systematic studies are required to unambiguously verify the origin of LEM.

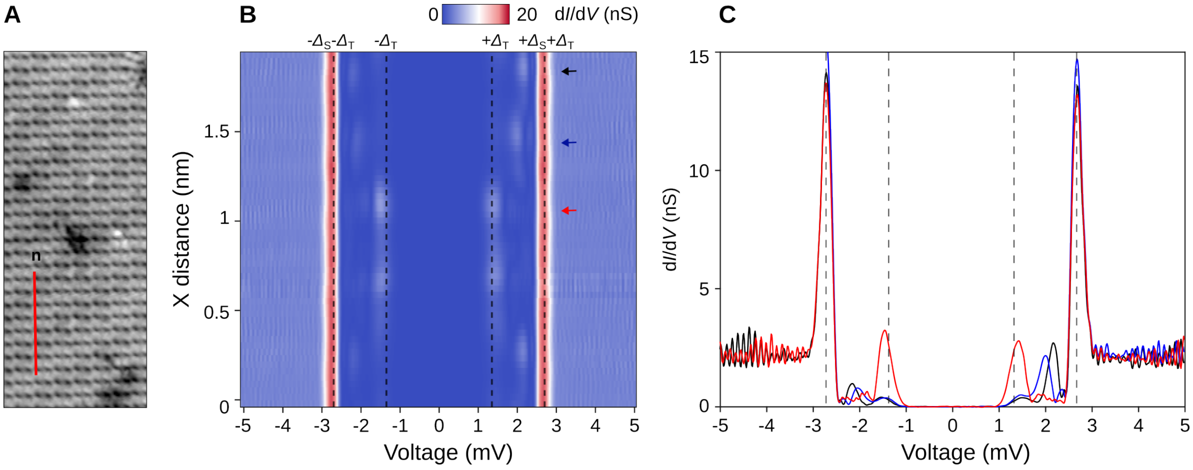

Figure S13: dI/dV measurements of neutral TBTAP^0 in n rows. (A), STM image of the TBTAP assembly. (B), dI/dV cross-section acquired along eight molecules of a n row at 1 K with a superconducting Pb tip. Dashed lines lines correspond to $\pm$ $(\Delta_T + \Delta_S)$ and $\pm$ Δ_T energies, respectively. (C), dI/dV point-spectra extracted at the black, blue and red arrows in (B). Lock-in parameters: f = 611 Hz, A_{mod} = 20 µeV.

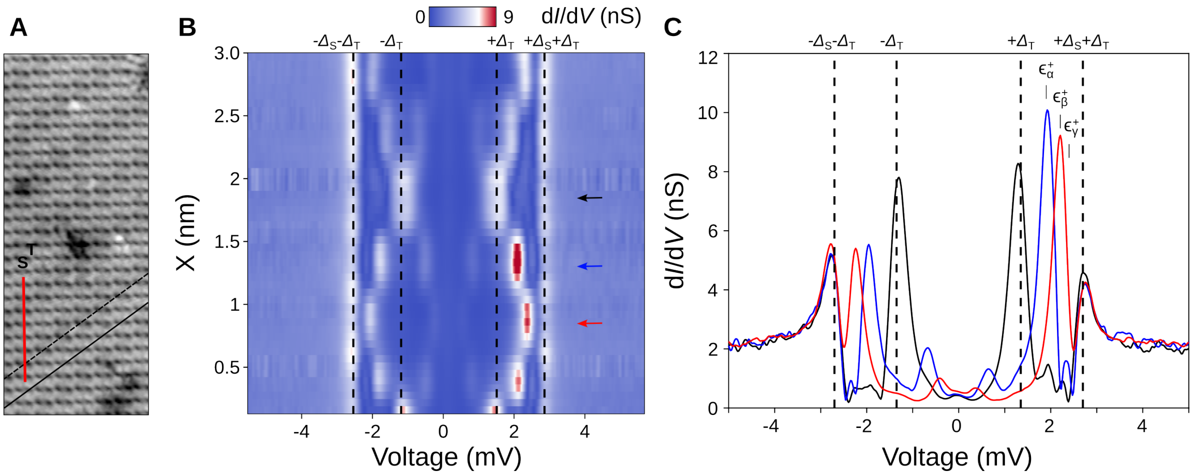

Figure S14: Yu-Shiba-Rusinov states along a c row at 4.2 K with a superconducting tip. (A), STM topographic image of the TBTAP supramolecular assembly on Pb(111), (I_t = 1 pA, V_s = 20 mV). (B), dI/dV cross-section taken along seven molecules of a c row (red line in A). Dashed lines lines correspond to $\pm$ $(\Delta_t + \Delta_s)$ and $\pm$ Δ_t energies, respectively. (C), dI/dV point-spectra extracted at the red, blue and black arrows of (B).

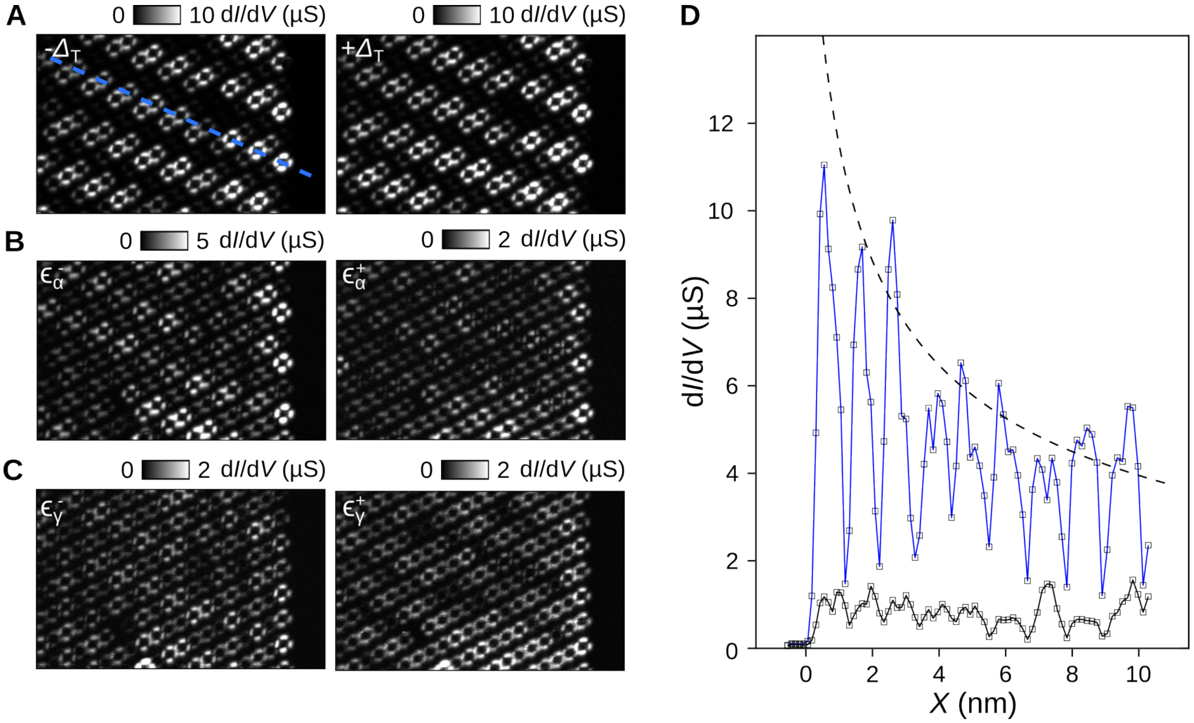

Figure S15: Localization length of LEM extracted from $\mathrm{d}I/\mathrm{d}V$ grid-spectroscopy. (A-C), Additional $\mathrm{d}I/\mathrm{d}V$ maps acquired at 1 K with a Pb tip at energies of $\pm\Delta_{\mathrm{T}}$, $\pm\varepsilon_\alpha$ and $\pm\varepsilon_\gamma$. (D), Extracted decay length of the LEM wavefunction (blue) from the grid spectroscopy of Fig. 3G acquired along the blue dashed line of (A). The black curve corresponds to the ε^+ energy (gray). The island border is set at $X = 0$ nm. The dashed line is a fit of the decay length of the edge mode using two exponent function.

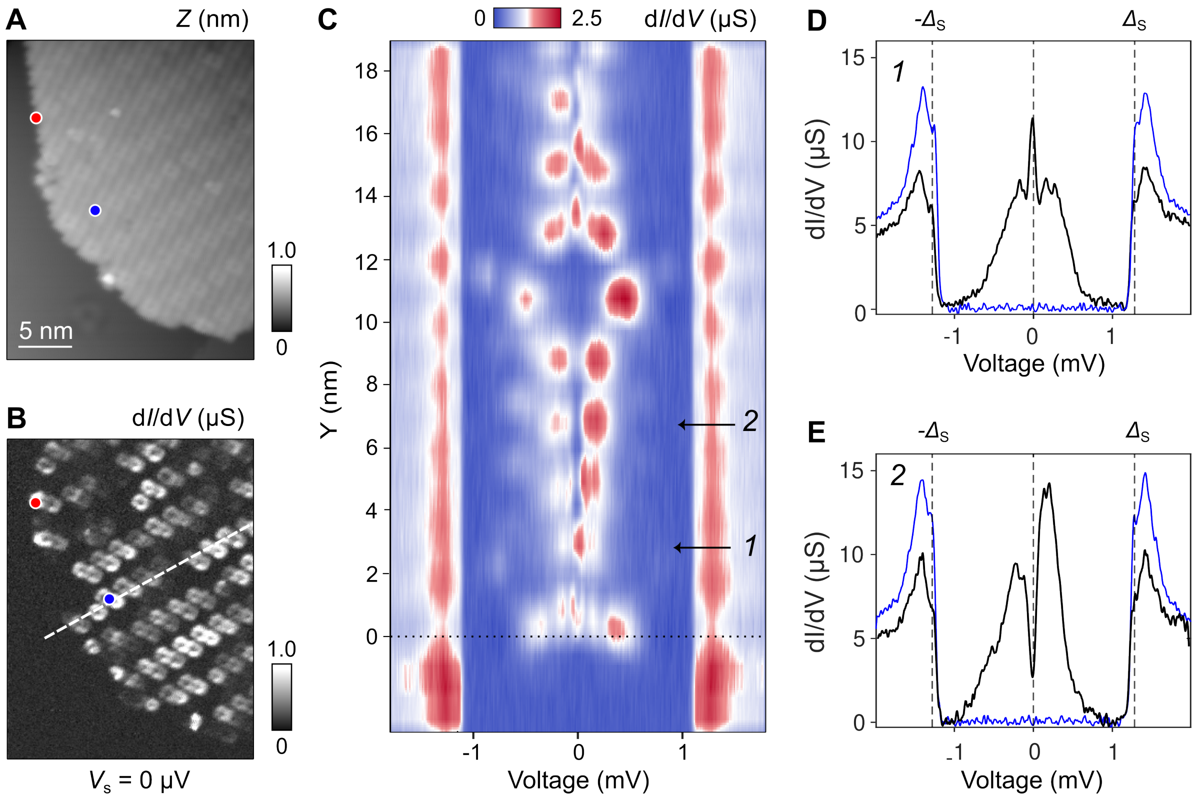

Figure S16: Spectral signatures of LEM at 50 mK with a metallic tip. (A), STM topography image of a TBTAP island on Pb(111). (B), $\mathrm{d}I/\mathrm{d}V$ maps at zero-energy showing the LEM lines. Lock-in parameters: $f = 3.2$ kHz, $A_{\mathrm{mod}} = 20$ μeV. The blue and red dots show the position of the $\mathrm{d}I/\mathrm{d}V$ spectra of Figure 4e and f of the main manuscript. (C), $\mathrm{d}I/\mathrm{d}V$(V,Y) cross-section acquired along the white dashed line in (B). The border of the island is set as $Y = 0$ nm and marked with doted lines. The blue curves show the $\mathrm{d}I/\mathrm{d}V$ spectra of the Pb(111) substrate. (D), Exemplary $\mathrm{d}I/\mathrm{d}V$ spectra extracted at the black arrows in (C).

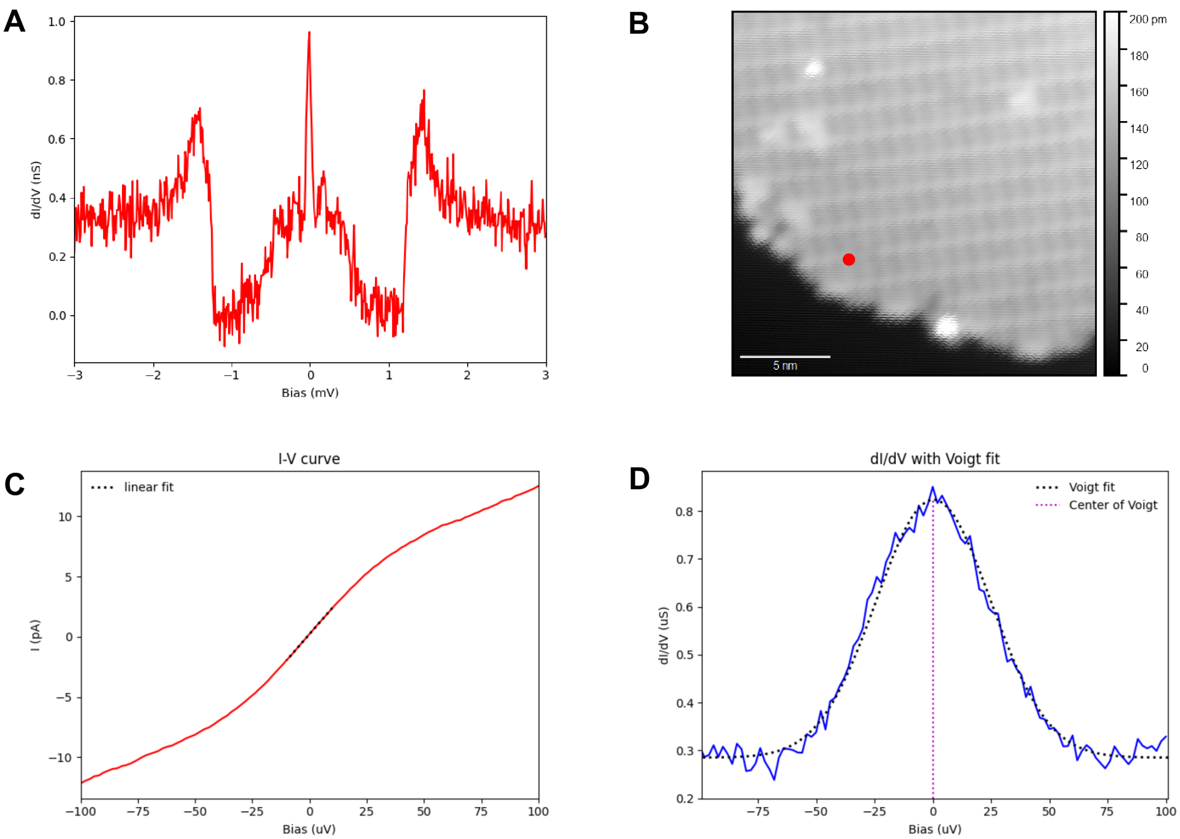

Figure S17: High-resolution $\mathrm{d}I/\mathrm{d}V$ spectra of the zero-energy peak. (A), $\mathrm{d}I/\mathrm{d}V$ single-spectra showing a sharp zero-energy peak. Lock-in parameters: $f = 3.08$ kHz, $A_{\mathrm{mod}} = 10$ µV, $T = 52$ mK, tunneling parameters: $I_{\mathrm{t}} = 200$ pA,$V_{\mathrm{s}} = 5$ mV. (B), STM topography image of a TBTAP island on Pb(111). The red dot shows the position of the spectra ($I_{\mathrm{t}} = 50$ pA, $V_{\mathrm{s}} = 300$ mV). (C), Current-voltage spectra of the ZBP. (D), Experimental $\mathrm{d}I/\mathrm{d}V$ spectra of the ZBP (blue). The black dashed line is a fit using a Voigt function. The Gaussian width $\sigma = 24.6\ \mu$V is consistent with the energy resolution of the STM while the additional Lorentzian width $\gamma = 2.47\times10^{-5}$ µeV is compatible with zero broadening of the ZBP. The small value of γ implies a large lifetimes of the ZBP. The center of the Voigt profile agrees with zero energy with an uncertainty of 0.3 µeV.

References

(1) Giessibl, F. J. The qPlus sensor, a powerful core for the atomic force microscope. *Rev. Sci. Instrum.* **2019**, *90*, 011101.

(2) Drechsel, C.; D'Astolfo, P.; Liu, J.-C.; Glatzel, T.; Pawlak, R.; Meyer, E. Topographic signatures and manipulations of Fe atoms, CO molecules and NaCl islands on superconducting Pb(111). *Beilstein J. Nanotechnol.* **2022**, *13*, 1–9.

(3) Balashov, T.; Meyer, M.; Wulfhekel, W. A compact ultrahigh vacuum scanning tunneling microscope with dilution refrigeration. *Rev. Sci. Instrum.* **2018**, *89*, 113707.

(4) Frisch, M. J. et al. Gaussian 16 Revision C.01. 2016; Gaussian Inc. Wallingford CT.

(5) Becke, A. D. Density-functional thermochemistry. III. The role of exact exchange. *J. Chem. Phys.* **1993**, *98*, 5648–5652.

(6) Kühne, T. D. et al. CP2K: An electronic structure and molecular dynamics software package - Quickstep: Efficient and accurate electronic structure calculations. *J. Chem. Phys.* **2020**, *152*, 194103.

(7) Perdew, J. P.; Burke, K.; Ernzerhof, M. Generalized Gradient Approximation Made Simple. *Phys. Rev. Lett.* **1996**, *77*, 3865–3868.

(8) Zhang, Y.; Yang, W. Comment on "Generalized Gradient Approximation Made Simple". *Phys. Rev. Lett.* **1998**, *80*, 890–890.

(9) Grimme, S.; Antony, J.; Ehrlich, S.; Krieg, H. A consistent and accurate ab initio parametrization of density functional dispersion correction (DFT-D) for the 94 elements H-Pu. *J. Chem. Phys.* **2010**, *132*, 154104.

(10) VandeVondele, J.; Hutter, J. Gaussian basis sets for accurate calculations on molecular systems in gas and condensed phases. *J. Chem. Phys.* **2007**, *127*, 114105.

(11) VandeVondele, J.; Krack, M.; Mohamed, F.; Parrinello, M.; Chassaing, T.; Hutter, J. QUICKSTEP: Fast and accurate density functional calculations using a mixed Gaussian and plane waves approach. *Comp. Phys. Comm.* **2005**, *167*, 103–128.

(12) Goedecker, S.; Teter, M.; Hutter, J. Separable dual-space Gaussian pseudopotentials. *Phys. Rev. B* **1996**, *54*, 1703–1710.

(13) Groth, C. W.; Wimmer, M.; Akhmerov, A. R.; Waintal, X. Kwant: a software package for quantum transport. *New J. Phys.* **2014**, *16*, 063065.

(14) Fernández-Torrente, I.; Franke, K. J.; Pascual, J. I. Vibrational Kondo Effect in Pure Organic Charge-Transfer Assemblies. *Phys. Rev. Lett.* **2008**, *101*, 217203.

(15) Fatayer, S.; Schuler, B.; Steurer, W.; Scivetti, I.; Repp, J.; Gross, L.; Persson, M.; Meyer, G. Reorganization energy upon charging a single molecule on an insulator measured by atomic force microscopy. *Nat. Nanotechnol.* **2018**, *13*, 376–380.

(16) Cockins, L.; Miyahara, Y.; Bennett, S. D.; Clerk, A. A.; Studenikin, S.; Poole, P.; Sachrajda, A.; Grutter, P. Energy levels of few-electron quantum dots imaged and characterized by atomic force microscopy. *Proc. Nat. Acad. Sci.* **2010**, *107*, 9496–9501.

(17) Mott, N. *Metal-Insulator Transitions*; CRC Press: London, 1990.

(18) Yao, N.; Glazman, L.; Demler, E.; Lukin, M.; Sau, J. Enhanced Antiferromagnetic Exchange between Magnetic Impurities in a Superconducting Host. *Phys. Rev. Lett.* **2014**, *113*, 087202.
